# Nervous Necrosis Virus-like Particle (VLP) Vaccine Stimulates European Sea Bass Innate and Adaptive Immune Responses and Induces Long-Term Protection against Disease

**DOI:** 10.3390/pathogens10111477

**Published:** 2021-11-12

**Authors:** Sofie Barsøe, Kerstin Skovgaard, Dagoberto Sepúlveda, Ansgar Stratmann, Niccolò Vendramin, Niels Lorenzen

**Affiliations:** 1 National Institute of Aquatic Resources (DTU AQUA), Technical University of Denmark, 2800 Lyngby, Denmark; sofhan@aqua.dtu.dk (S.B.); dsep@aqua.dtu.dk (D.S.); niven@aqua.dtu.dk (N.V.); 2 Department of Biotechnology and Biomedicine (DTU Bioengineering), Technical University of Denmark, 2800 Lyngby, Denmark; kesk@dtu.dk; 3 W42 Industrial Biotechnology GmbH, 44227 Dortmund, Germany; a.stratmann@w42biotechnology.de

**Keywords:** viral nervous necrosis, duration of immunity, virus-like particle, protective immunity, neutralizing antibodies, passive immunization

## Abstract

The rapidly increasing Mediterranean aquaculture production of European sea bass is compromised by outbreaks of viral nervous necrosis, which can be recurrent and detrimental. In this study, we evaluated the duration of protection and immune response in sea bass given a single dose of a virus-like particle (VLP)-based vaccine. Examinations included experimental challenge with nervous necrosis virus (NNV), serological assays for NNV-specific antibody reactivity, and immune gene expression analysis. VLP-vaccinated fish showed high and superior survival in challenge both 3 and 7.5 months (1800 and 4500 dd) post-vaccination (RPS 87 and 88, OR (surviving) = 16.5 and 31.5, respectively, *p* < 0.01). Although not providing sterile immunity, VLP vaccination seemed to control the viral infection, as indicated by low prevalence of virus in the VLP-vaccinated survivors. High titers of neutralizing and specific antibodies were produced in VLP-vaccinated fish and persisted for at least ~9 months post-vaccination as well as after challenge. However, failure of immune sera to protect recipient fish in a passive immunization trial suggested that other immune mechanisms were important for protection. Accordingly, gene expression analysis revealed that VLP-vaccination induced a mechanistically broad immune response including upregulation of both innate and adaptive humoral and cellular components (*mx*, *isg12*, *mhc I*, *mhc II*, *igm*, and *igt*). No clinical side effects of the VLP vaccination at either tissue or performance levels were observed. The results altogether suggested the VLP-based vaccine to be suitable for clinical testing under farming conditions.

## 1. Introduction

The European sea bass (*Dicentrarchus labrax*) is one of the main cultured species in the Mediterranean region, with increasing production every year [1]. The production cycle takes two to three years until the plate-sized sea bass are harvested. During the production cycle, sea bass are exposed to various pathogens. One of these is red-spotted grouper nervous necrosis virus (RGNNV), which causes viral nervous necrosis (VNN) or viral encephalo- and retinopathy. The disease is characterized by necrosis of nervous tissues and a spiraling swimming pattern. VNN may result in high mortalities—particularly at the larval stage, where up to 100% mortality has been reported [2,3,4,5]—and is therefore considered the main viral pathogen of concern for the sea bass production [6]. RGNNV belongs to the genus of *Betanodavirus*, which are small (~30–35 nm), icosahedral, nonenveloped, single-stranded, positive-sense RNA viruses with a bisegmented genome [7]. RNA1 encodes the RNA-dependent polymerase, and RNA2 encodes the capsid protein (CP), which consists of an N-terminal arm, a shell domain, and a protrusion domain [8]. Nervous necrosis virus (NNV) is divided into four species, which together infect more than 120 marine and freshwater species [7,9]. The species RGNNV generally infects warm-water fish such as European sea bass in the Mediterranean and Asian sea bass (*Lates calcarifer*), orange spotted grouper (*Epinephelus coioides*), and dragon grouper (*E. lanceolatus*) along the coast of Asia and around Australia [9].

The disease is preventable through vaccination, and several experimental VNN vaccines have been shown to induce neutralizing antibodies and protect against disease in experimental challenges [10]. Recently, two commercial vaccines were registered for use in selected Mediterranean countries [11,12]. These vaccines were based on inactivated virus particles and were formulated with either a mineral or nonmineral oil adjuvant to enhance the immune response. One was registered to provide immunity for one year, and for the other, duration of immunity was not specified. In addition, different experimental VNN vaccines have been studied, including inactivated virus vaccines, recombinant capsid protein vaccines, DNA vaccination, and vaccines based on virus-like particles (VLPs) [13,14,15,16,17]. 

The NNV VLP-based vaccines consist of recombinant viral CP, which is produced in an expression host system following insertion of a DNA fragment corresponding to the NNV RNA2 sequence into a host vector (usually plasmid or virus) or directly into the host genome. After transformation, the host cells express the CP, which auto-assembles into VLPs intracellularly. The VLPs can subsequently be released by breakage of the host cells and then be purified by cost-efficient techniques such as differential precipitation or centrifugation [18]. Although resembling the virus, the VLPs lack the viral genome and thus are unable to infect and replicate in the fish host. In general, VLPs have demonstrated great potential as prophylactic vaccines against several viral diseases, such as human papilloma, human hepatitis B, malaria, porcine circovirus type 2 and recently also the pandemic COVID-19 (reviewed by [19,20,21]). The immunogenic size (30–35 nm) of VLPs along with their virus-like structure is probably the background for their efficient recognition by the immune system, which stimulates strong and long-lasting humoral and cellular responses [19,21,22]. Compared to inactivation or attenuation of virus, VLPs contain no remnants of the chemical used for inactivation, nor is there a chance of lack of inactivation or return to virulence. Furthermore, in an optimal expression vector, a high production yield of VLP can be achieved with minimal costs [18].

Thiéry et al. [14] produced an RGNNV VLP in a baculovirus expression vector and demonstrated that the VLP elicited dose-dependent protection in European sea bass when administered intramuscularly (IM) in fish that were then challenged with live RGNNV IM one month after immunization. Likewise, an RGNNV VLP produced in *E. coli* [23] induced a strong humoral response in dragon grouper and Malabar grouper (*E. malabaricus*) when administered IM [24]. Lai et al. [25] further investigated this VLP as a vaccine candidate in Asian sea bass; they demonstrated an upregulation of immune-related genes in addition to a high antibody response.

We recently presented results on a novel RGNNV VLP produced in the yeast *Pichia pastoris* (*Komagataella phaffii*). This VLP-based vaccine was administered to European sea bass intraperitoneally and protected against disease caused by RGNNV in experimental challenges at one and two months post-vaccination (pv) (570 and 1180 degree days (dd) pv, respectively) [26]. Elevated survival was also seen following challenge at 10 months pv, but low temperature during immunization was suspected to compromise the immune response [26]. In the present work, we further investigated the long-term protection induced by the VLP-based vaccine in an optimized and improved experimental setup for almost 9 months (5000 dd) with viral challenges by either bath or IM injection at 3 and 7.5 months pv (1700 and 4500 dd, respectively). The induced immune response was characterized through the detection of NNV/VLP-reactive antibodies in the serum of vaccinated fish and by gene expression analysis of 19 key immune genes in various tissues from vaccinated fish. Furthermore, we examined the role of antibodies in the protection in a passive immunization experiment in which naïve sea bass recipients were injected with donor serum from larger, hyperimmunized sea bass prior to NNV challenge.

## 2. Results

### 2.1. Morphology and Antigenicity of the VLP

**Figure 1:**
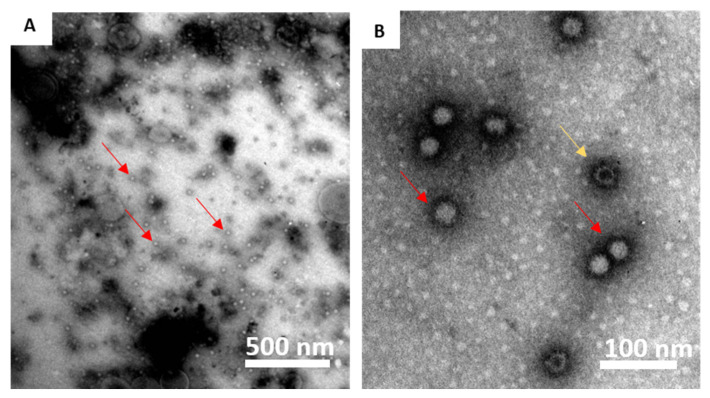
Transmission electron microscopy of the virus-like particles (VLPs) used in the experiments. Magnification: (**A**) 14,000×; (**B**) 110,000×. Some VLPs appeared to be packed, presumably with cytoplasmic material from the Pichia host cells (red arrows), while others were empty (yellow arrow). Photo credit: Istituto Zooprofilattico Sperimentale Venezie (IZSVe, Padua, Italy).

**Figure 2:**
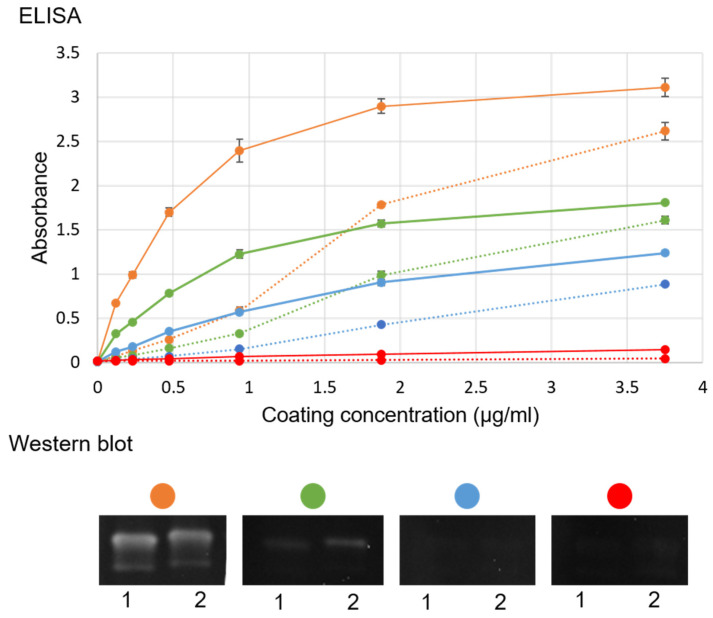
Reactivity of serum against live RGNNV and RGNNV VLPs produced in *Pichia pastoris.* Polyclonal rabbit-anti-noda serum [27] (dil 1:3000 in ELISA and 1:1000 in Western blot) (brown); serum from sea bass (dil 1:200) hyperimmunized with either RGNNV VLP (green), commercial vaccine (blue), or PBS (red). ELISA coated with different concentrations of either VLP (straight line) or ultracentrifuged RGNNV (dotted line) and incubated with fish serum or polyclonal rabbit-anti-noda serum. Each serum/coating combination was made in duplicate wells, and the results presented here are the duplicates’ means of absorbance at 450 nm minus the absorbance at 620 nm. Error bars indicate the standard error of duplicates. Western blot of ultracentrifuged RGNNV virus (160 µg/mL) (**1**) and RGNNV VLPs (80 µg/mL) (**2**) with different primary antibodies (according to color).

The VLPs were previously characterized [26], although since this was a new production batch, the morphology was confirmed via transmission electron microscopy (Figure 1), and antigenicity was compared to ultracentrifuged RGNNV virus via Western blot (WB) (Figure S1 and Figure 2).

### 2.2. Comparison of Antibody Reactivity with VLP and RGNNV 

Sera from hyperimmunized sea bass were tested for specific reactivity in ELISA coated with different concentrations of ultracentrifuged RGNNV or VLP and in WB with the same samples. Specific antibody reactivity was evident against both RGNNV and VLP, although a stronger signal was seen in the plates coated with VLP and for sera from fish immunized with the commercial vaccine (Figure 2). Therefore, it was decided to use VLP as coating in the ELISA protocol from that point onwards.

### 2.3. Vaccination with the VLP-Based Vaccine Was without Side Effects

Intraperitoneal injection with the VLP solution did not result in mortality in the first 21 days after vaccination (Table S1) or any notable side effects. The mean weight of the VLP-vaccinated fish was not significantly different from that of the mock-vaccinated fish at any timepoint (Table S1). The fish receiving the commercial (COM) vaccine weighed less than the mock-vaccinated fish, although not significantly less. However, the VLP-vaccinated fish weighed 27.5% (9.4–45.7%/3.8 g (1.3–6.3 g)) more than the COM-vaccinated fish at T1 and 44.6% more (7.8–81.9%/16.5 g (2.9–30.3 g)) at T2 (*p* < 0.05 (ANOVA), Table S1). The fish were not weighed prior to the challenge to minimize handling stress, which could possibly influence survival.

### 2.4. The VLP-Based Vaccine Induced a Long-Lasting Response of Specific and Neutralizing Antibodies

**Figure 3:**
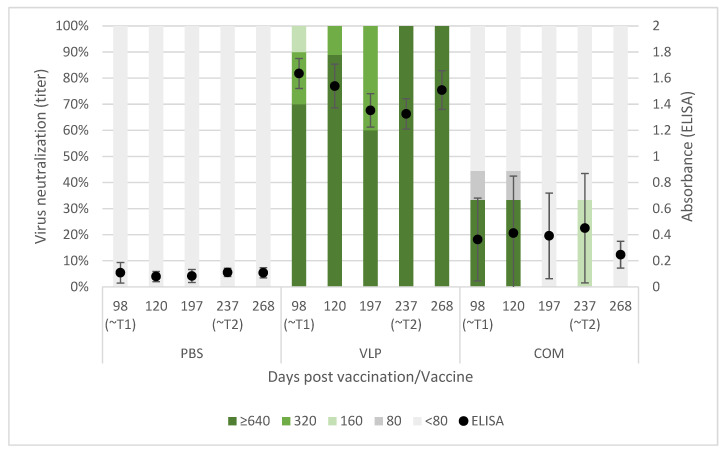
Specific antibody reactivity in ELISA and virus-neutralizing activity in sera from vaccinated sea bass. Left axis: Proportion with the given neutralizing titer. The titer is expressed as the reciprocal of the dilution providing inhibition of cytopathic effect in both wells at day 8 postinoculation. Right axis: ELISA results of the same samples. The absorbance was measured at wavelengths 450 nm and 620 nm. The plot represents the mean absorbance (Abs_450 nm_ – Abs_620 nm_) ± standard deviation (*n* = 5, 9, 5, 6, 6, 10, 9, 5, 6, 6, 9, 9, 5, 6, 6 (order as in Figure)).

Specific IgM antibodies against NNV were detected throughout the experimental period of 268 days (~9 months/5000 dd) by ELISA (Figure 3) in serum from all sampled VLP-vaccinated fish. Furthermore, the virus-neutralizing activity of this serum was demonstrated with titers ranging from 160 to >640. Specific and neutralizing antibodies were also demonstrated in serum samples from fish receiving the COM vaccine, but not in all samples and in general at lower levels in ELISA and with a smaller proportion of the sampled sera showing neutralizing activity, decreasing over time (Figure 3). Neither antibody reactivity with VLP antigen nor virus-neutralizing serum activity was demonstrated in the mock-vaccinated fish receiving PBS (Figure 3).

### 2.5. VLP-Vaccinated Fish Were Protected against Viral Disease in Bath and Injection Challenge

**Figure 4:**
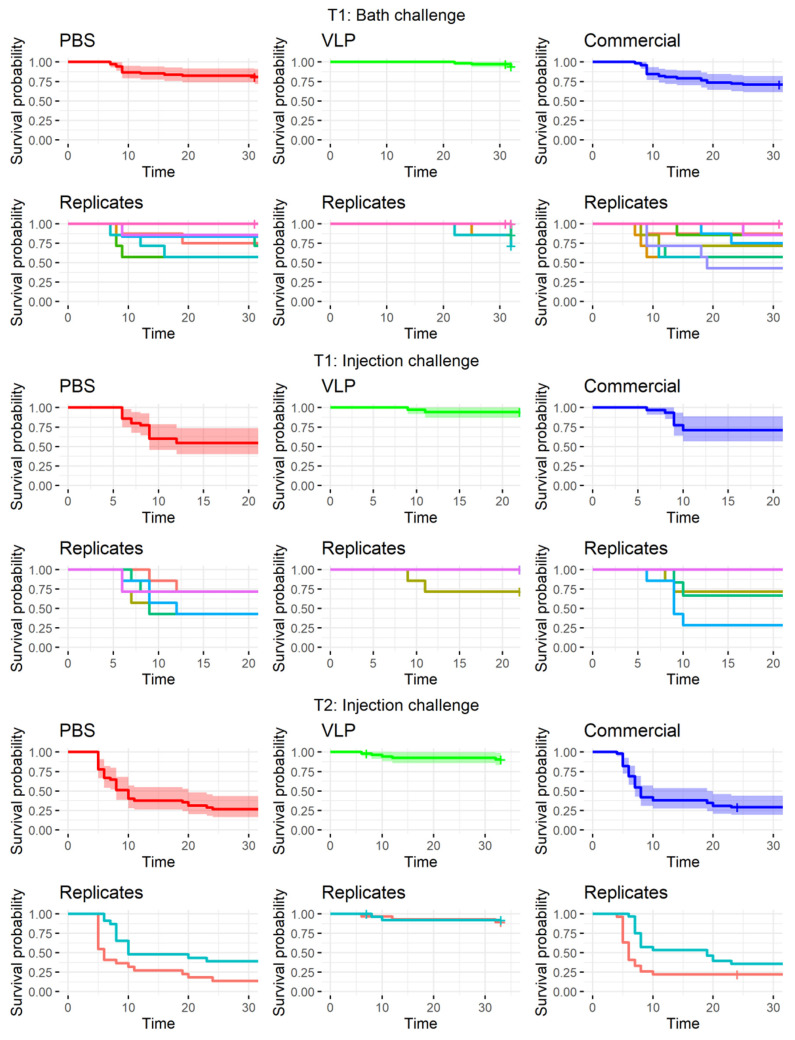
Survival curves of the pooled data for each treatment and for the replicate tanks at each challenge timepoint/route: T1 bath and T1 injection 93 days (~3 months/1800 dd) after vaccination and T2 injection 240 days (~7.5 months/4500 dd) after vaccination. Temperature data from each challenge is available in Figures S2–S5. PBS = mock vaccination with phosphate-buffered saline, VLP = vaccination with RGNNV virus-like particles, Commercial = vaccination with a commercial vaccine.

The viral challenges were successful, with fish developing clinical signs around day 4–6 postinfection (Figure 4, Video S1). The VLP-vaccinated fish had >90% survival and a higher survival than the mock-vaccinated controls in all the challenges (Table 1, Figure 4). In the injection challenges, the VLP-vaccinated fish had 16.5 and 31.5 times higher chances of surviving than the mock-vaccinated fish at T1 and T2, respectively (T1: *p* < 0.01 and T2: *p* < 0.001 (Table 2)). Moreover, at the T2 challenge 7.5 months after vaccination, the VLP-vaccinated fish also had 30 times higher chance of surviving than the fish receiving the COM vaccine (*p* < 0.001, Table 2). In the bath challenge, considerable variability between replicate tanks was observed. When pooling the individual fish from each tank into one dataset, almost 20% of the mock-vaccinated fish were recorded as diseased (Table 1). Only 5.7% of the VLP-vaccinated fish developed disease, but the difference from the mock-vaccinated fish was at the borderline in terms of significance (*p* = 0.057, Table 2). The fish receiving the commercial vaccine had the lowest survival, which was significantly lower than that of the VLP-vaccinated fish (*p* < 0.01, Table 2), although not significantly different from that of the mock-vaccinated fish. The diseased fish in the group vaccinated with the commercial vaccine and challenged via bath had a characteristic clinical appearance different from that of the other groups after being challenged by bath This was characterized by intense inflammation and hemorrhage in the nasal cavity, very dark coloration of the skin, and lethargic swimming (Video S1). This clinical appearance was different from that observed in the other two groups, wherein the main clinical signs were a spiraling swimming pattern and sometimes dark coloration of the skin.

### 2.6. Confirming the Cause of Disease 

The cause of disease was determined to be NNV by the characteristic clinical signs and by detection or isolation of the virus from diseased fish. A representative number of fish from all groups/tanks were sampled for isolation of the virus. All moribund fish from injection challenges and all investigated COM and PBS fish from the bath challenge were positive for viral RNA or live virus. In the bath challenge, only four VLP-vaccinated fish succumbed to the infection relatively late after the challenge (on day 22–32 post-challenge). Three of these were analyzed by reverse transcription (RT) real-time quantitative polymerase chain reaction (qPCR), and the brain from the fourth fish was used to isolate the live virus on an SNN1 cell culture. Two of the three RTqPCR analyses were found negative for viral RNA despite normal amplification of the reference gene mRNA, confirming that RNA was successfully extracted and of sufficient quality/integrity. From the fourth fish, live virus was successfully isolated. 

### 2.7. Censoring

At T1, one of the replicate bath-challenged tanks (replicate X, Table 1) did not have any fish developing clinical signs during the experimental period (32 days), although viral RNA was detected in two out of three analyzed survivors, indicating that the fish were indeed infected, although probably only at a low level. This bowl was still included in the results. In two of the replicate tanks at T1 with fish challenged by injection, problems with air supply resulted in acute loss overnight of more than half of the fish. These two replicates were removed from the dataset for that reason and are not included in Table 1. At day 22, another tank (replicate III) experienced a similar technical issue, and five fish were lost (2 × VLP, 2 × PBS and 1 × COM). As it had been 7 days since the last VNN-related euthanasia, it was decided to terminate the injection challenge experiment at day 22 and count the remaining fish as survivors. During the T2 challenge, two fish jumped out of the tanks during cleaning (one VLP and one COM). These were both removed from the dataset.

### 2.8. Vaccination Reduced the Number of Survivors with Detectable Viral RNA or Virus

**Figure 5:**
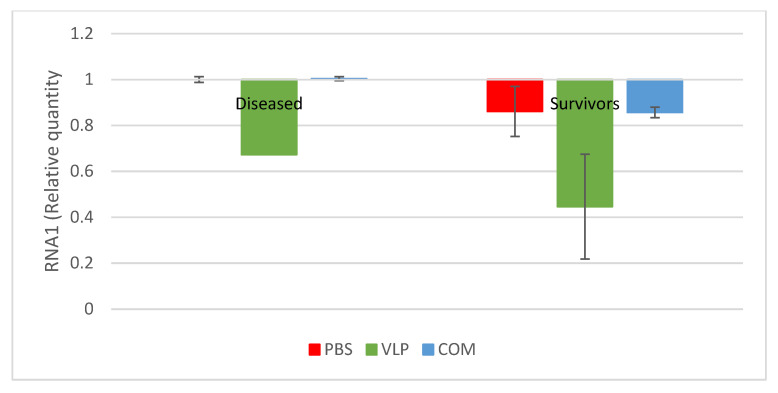
Relative amounts of viral RNA in brains of diseased and survivors in T1 bath challenge (3 months post-vaccination). Only positive samples included. Scaled to the mean of the PBS-diseased group (Amount of virus in PBS-group = 1). Error bars indicate the standard error of the mean (SEM) (*n* = 6, 1, 9, 8, 3, 6 (order as in figure)).

Fewer VLP-vaccinated survivors were positive for virus in their brain and/or organs (Table 3). In the T1 bath and injection challenge, there were significantly fewer VLP-vaccinated survivors testing positive for viral RNA in RTqPCR (Table 3). The level of RNA in the few positive samples were also evaluated, and there was neither a significant difference between diseased and survivors nor between the groups (Figure 5). The low number of positive samples among the VLP-vaccinated survivors made the analyzed numbers low in this group (*n* = 2 in injection challenge and *n* = 3 in bath challenge), compromising statistical analysis.

### 2.9. Serology of Survivors

**Figure 6:**
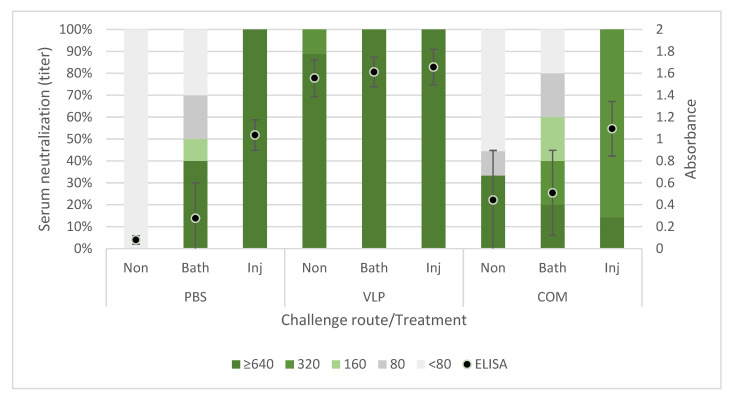
Specific and neutralizing activity of serum from survivors in T1 challenge, sampled 120–124 days after vaccination (28–32 days after challenge). Non = mock challenged, Bath = bath challenged, Inj = injection challenged. Left axis: Proportion with the given neutralizing titer. The neutralizing titer is expressed as the reciprocal of the dilution providing inhibition of cytopathic effect in both wells at day 8 postinoculation. Right axis: ELISA results of the same samples (dil 1:200). The absorbance was measured at the wavelengths of 450 nm and 620 nm. The plot represents the mean absorbance (Abs_450nm_ − Abs_620nm_) ± standard deviation (*n* = 9,10,6,9,10,7,9,10,7 (order as in figure)).

All the survivors from experimental challenge (T1) had an increase in specific antibodies, measured via ELISA. Furthermore, an increase in the proportion of fish with neutralizing antibodies, as well as an increase in titer of these, was seen. This increase was highest in the fish surviving the injection challenge (Figure 6). 

### 2.10. Immune Gene Expression

**Figure 7:**
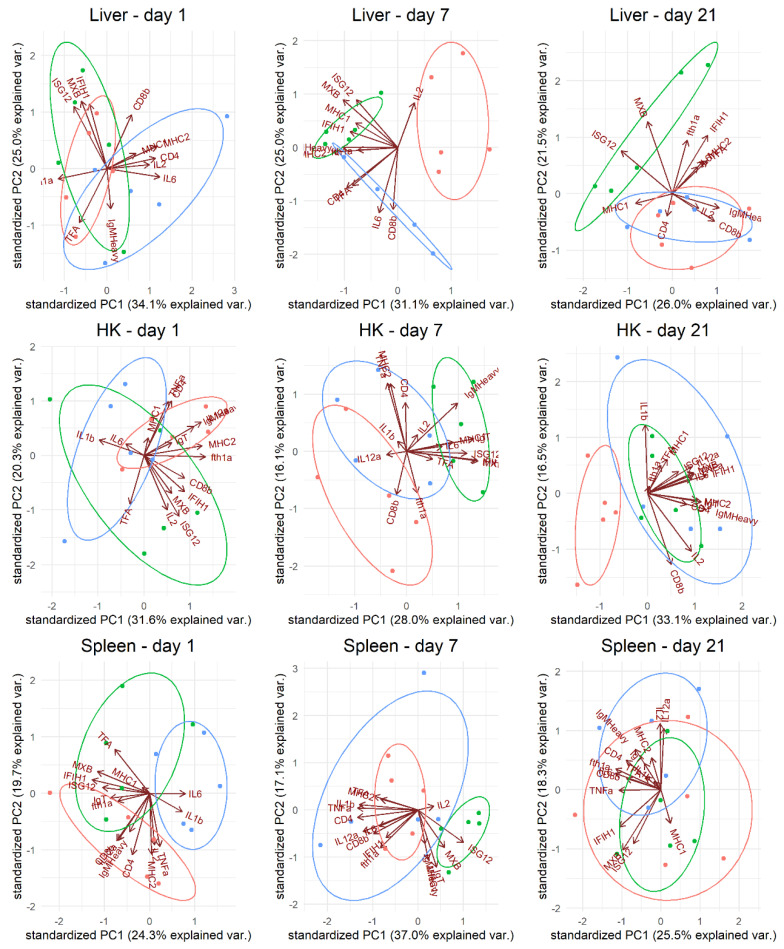
Principal component analysis of gene expression in different tissues (liver, head kidney (HK), and spleen) on days 1, 7, and 21 pv from fish vaccinated with either VLP-based vaccine (green), commercial vaccine (blue), or mock vaccinated with PBS (red). The vectors represent the influence of each gene.

**Figure 8:**
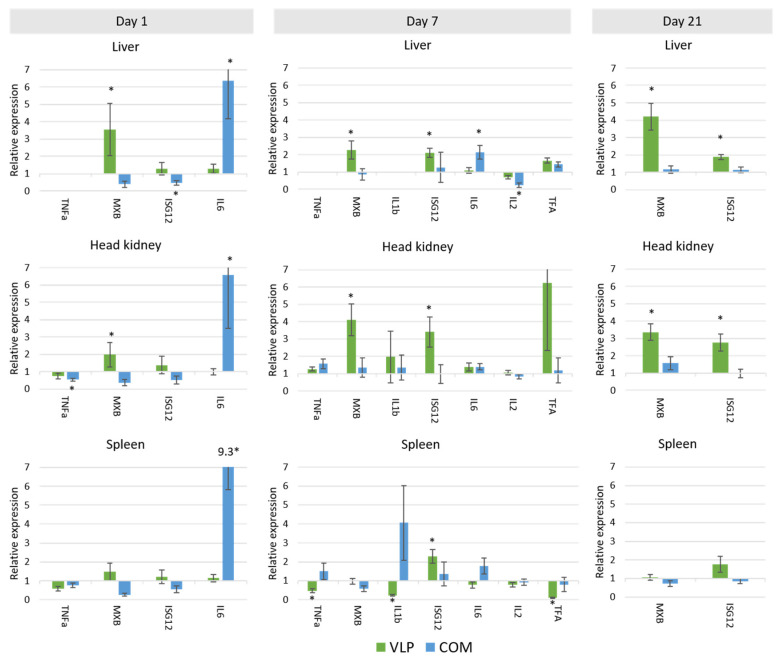
Expression of mRNA of immune genes involved in innate immunity in European sea bass vaccinated with either VLP vaccine (VLP) or commercial vaccine (COM). The expression is plotted as a relative expression to the mRNA level in mock-vaccinated fish sampled on the same timepoint. Missing bars mean that mRNA was not expressed in the given tissue. * = biologically relevant (significantly different from mock-vaccinated fish (*p* < 0.05, *t*-test) and more than double up- or downregulated compared to the mock-vaccinated fish). Data for all genes are available in Supplementary Table S3.

**Figure 9:**
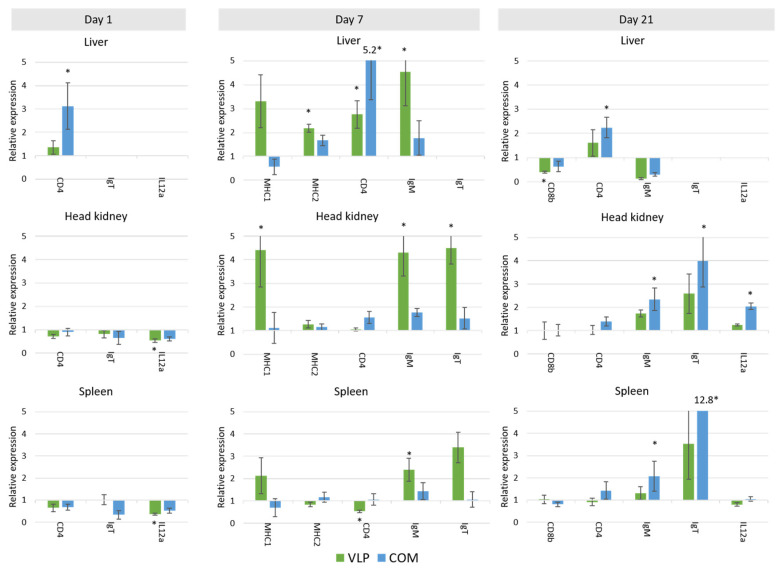
Expression of mRNA of immune genes involved in adaptive immunity in European sea bass vaccinated with either VLP vaccine (VLP) or commercial vaccine (COM). The expression is plotted as a relative expression to the mRNA level in mock-vaccinated fish sampled on the same timepoint. Missing bars means that the mRNA was not expressed in the given tissue. * = biologically relevant (significantly different from mock-vaccinated fish (*p* < 0.05, *t*-test) and more than double up- or downregulated compared to the mock-vaccinated fish). Data for all genes are available in Supplementary Table S3.

Most of the selected immune genes were expressed in all tissues, although not in all samples. *ifn* and *cox-2* expression were not detected in high enough amounts to be quantified. At least two reference genes in each tissue were validated as suitable for normalization (Table S2). In all tissues, *18srrna* was expressed too much (i.e., Cq values were too low) to be used as a reference gene. In the principal component analysis (PCA) of the gene expression, clustered expression patterns could be recognized based on the kind of vaccine used (PBS, VLP, and/or COM) on day 1 (spleen), day 7 (liver, head kidney (HK), spleen), and day 21 (HK, liver) (Figure 7). Both up- and downregulations were observed in VLP-vaccinated fish compared to mock-vaccinated fish (Figure 8 and Figure 9). In the HK and liver, significantly increased expression of *mx* in VLP-vaccinated fish occurred already at day 1. This continued at days 7 and 21 in the HK and liver while supplemented by a significant increase in *isg12*, *mhc II*, *CD4*, and *igm* in the liver and *mhc I*, *igt*, and *igm* in the HK on day 7. In the spleen, a downregulation of *il12*, *igt*, and *mx* were seen on day 1 in VLP-vaccinated fish, though this was significant only for *il12*. This was followed by a downregulation of *tnfa*, *il1b*, *tfa*, and *cd4* on day 7, while only *isg12* and *igm* were significantly upregulated in the spleen. A different response profile was seen in the group receiving the commercial vaccine, where significant expression of *il6* and *cd4* mRNA was detected in the liver on day 1 and 7, while only *cd4* was upregulated on day 21 in the liver. In the HK, increased expression of *igt* and *igm* was seen on day 21, on which day the VLP-vaccinated fish did not show a significant increase. 

Few gene expression differences were seen in the brains of VLP- or COM- compared to mock-vaccinated animals. However, on day 7, there was significantly higher expression of *mxb* and *isg12* in the brains of the VLP-vaccinated (1.1 (SEM = 0.2) and 2.1 (SEM = 0.5), respectively) compared to the COM-vaccinated fish (0.5 (SEM =0.1) and 0.6 (SEM = 0.1), respectively) (data not shown).

### 2.11. Passive Immunization

**Figure 10:**
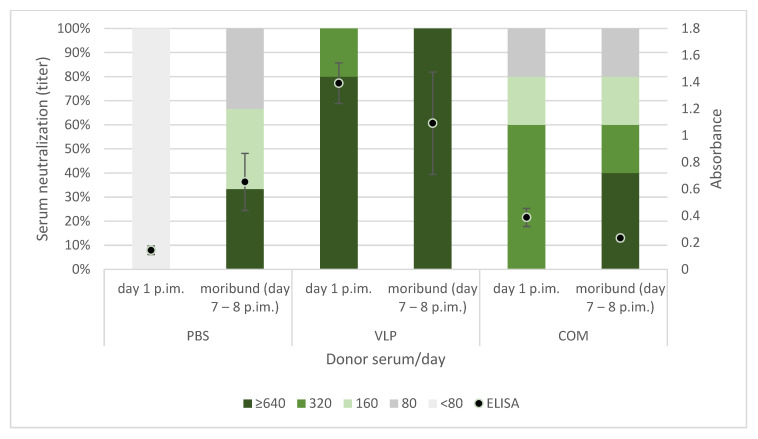
RGNNV specific antibodies in serum from passively immunized recipient sea bass on day 1 and 7 or 8 post immunization (p.im.). Samples from day 1 were sampled before challenge, and samples from day 7–8 were from infected moribund recipient fish (challenged at day 1). Left axis: Proportion with the given neutralizing titer. The neutralizing titer is expressed as the reciprocal of the dilution, providing inhibition of cytopathic effect in both wells at day 8 postinoculation. Right axis: ELISA results of the same samples (dil 1:200). The absorbance was measured at the wavelengths of 450 nm and 620 nm. The plot represents the absorbance (Abs_450nm_ − Abs_620nm_) ± standard deviation. (*n* = 3, 3, 5, 5, 5, 5, 5, 5, 5 (order as in figure)).

The recipient sea bass were successfully passively immunized with serum from the larger sea bass hyperimmunized with either VLP-based vaccine, commercial vaccine, or PBS (VLP-, COM- or PBS-donor serum). RGNNV-specific antibodies were detected in the recipients on day 1 (day of challenge) and in infected moribund recipient fish on day 7 or 8 postimmunization, sampled before termination (Figure 10). Interestingly, in recipient moribund fish, significant increases in specific antibodies were seen only in the fish that had received PBS-donor serum (*p* < 0.01, *t*-test). 

**Figure 11:**
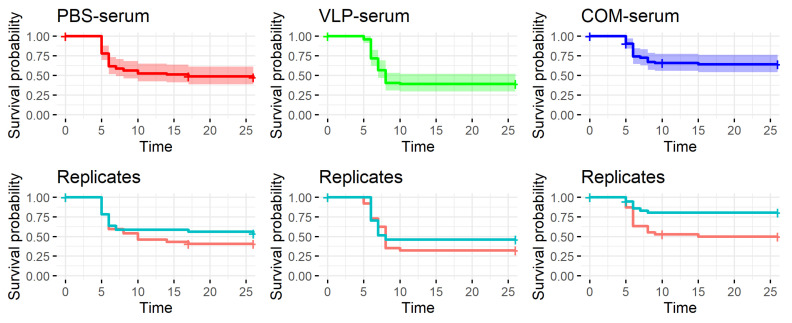
Survival after experimental challenge of sea bass passively immunized with serum from donor fish hyperimmunized with either phosphate-buffered saline (PBS-serum), virus-like particle vaccine (VLP-serum), or commercial vaccine (COM-serum). The curves in the first row represent the pooled data from both tanks. The shaded areas indicate the 95% confidence intervals of the survival probabilities. In the second row, the survival in the two replicate tanks are visualized. Time = days post-challenge.

The fish were challenged one day after receiving the serum. At day 5 postinfection, the first fish with clinical signs were observed and euthanized (Figure 11). The recipients of the VLP-donor serum had the lowest survival in both replicate tanks. The recipients of the commercial-donor serum seemed to have the highest survival, although with considerable variation between the two replicate tanks (Table 4 and Figure 11). There was no significant difference between the survival in the PBS-serum recipients and the two groups receiving positive serum (*p* > 0.05, “VLP–PBS”, “PBS–COM”, Table 5).

## 3. Discussion

To protect farmed sea bass in the Mediterranean aquaculture against viral nervous necrosis, an effective vaccine providing long-lasting protection is needed. We here tested a promising VLP-based vaccine in a time-course study with challenges at 3 and 7.5 months after vaccination (1700 and 4500 dd). The VLP-based vaccine induced a high response of specific and neutralizing antibodies lasting at least until 9 months (5000 dd) pv, even without formulation with an adjuvant. More importantly, the VLP-vaccinated fish were protected against VNN in experimental challenges at 3 (T1) and 7.5 (T2) months pv with significantly higher odds of surviving than mock-vaccinated fish (T1: OR = 16.5 (*p* < 0.01) and T2: OR = 31.5 (*p* < 0.001)). This underlines the ability of the VLP-vaccine to induce long-term protection, which is pivotal for the applied potential of the vaccine. The induced antibody response and protection did not seem to decline within the examined period (~9 months/5000 dd). These results confirmed and added to our previous studies, wherein the VLP-based vaccine also protected sea bass in experimental injection challenges at 1 and 2 months pv (570 and 1180 dd, respectively) [26]. In that experiment, very low protection was seen 10 months (3600 dd) pv. The reduced long-term protection reported in the previous study could most likely be explained by a high challenge viral dose and/or a too low rearing temperature (12 °C) impairing the immune response. The adaptive immune response in teleost fish is temperature dependent, and the optimal water temperature for antibody development in sea bass is 24 °C [29]. Previous studies with NNV VLP vaccination of sea bass also found a protective effect in experimental challenge ~30 days (675–875 dd) pv [14,30,31]]. This has also been demonstrated in other marine species such as grouper (*Epinephelus* ssp.) [32,33] and turbot (*Scopthalmus maximus*) [13] 21 to 63 days (525–1400 dd) pv. To our knowledge, the current study is the first to demonstrate long-term protection against VNN at 7.5 months (4500 dd) after VLP vaccination and also the first study reporting a corresponding long-lasting antibody response.

Intraperitoneal injection with the purified VLP did not cause any increase in mortality during the observation period of 21 days, and the injection did not negatively influence growth during the 9 months that the fish were in the experiment. On the contrary, the co-habitant fish receiving the commercial vaccine seemed to have reduced growth. Possibly, the adjuvant in the commercial vaccine, designed to induce local inflammation for efficient activation of the immune system, caused the vaccinated fish to have reduced appetite for a short period, as also seen in salmonids [34,35]. Accordingly, we also saw signs of inflammatory reactions such as tissue adherences and melanocyte infiltrations in the peritoneal cavity only among fish given the commercial vaccine (unpublished observations). The commercial vaccine used here was registered for use in sea bass >12 g and might have been harder to tolerate for the smaller fish in our experiment (5 ± 2 g); this could potentially have contributed to the weaker antibody response and lower protection. However, similar differences were observed earlier in sea bass vaccinated at 29 and 14 g [26]. Concerning the VLP-based vaccine, our results demonstrated that it was safe to administer to fish already from 5 g without delaying their growth, thereby potentially allowing earlier use than the currently available commercial vaccines.

Compared to a farming environment, the injection challenge represents an unnatural route of exposure. Several natural entry points of NNV has been proposed and confirmed, including the eyes/n. opticus, nasal cavity, epithelial cells on the body and/or fins, or ingestion [36], although no primary route has been confirmed. Thus, to mimic a more natural exposure to the virus, we also performed bath challenge at the first challenge timepoint (T1). However, previous studies using RGNNV (283.2009) in bath challenges have caused clinical disease in only 30–35% of naïve fish [5,37]. This typically increases tank-related variability and is also considered too low to demonstrate the potency of a vaccine according to the recommendations given by Amend [28]. As IM challenge has already been used in previous experiments to assess the efficacy of VLP vaccines in sea bass with satisfactory disease incidence [14,26], an injection challenge was performed in parallel, although more replicates were devoted to the bath challenge to strengthen the statistical analysis. We observed clinical signs in only 19.1% of the mock-vaccinated bath-challenged fish (Table 1), which was lower than expected, based on the mentioned literature. Consequently, we were not able to prove significant protection of the VLP-vaccinated fish in the bath challenge, even though only 5.7% of these developed clinical disease, and this occurred later in the challenge period compared to the mock-vaccinated controls (Figure 4). In addition, significantly fewer of the analyzed VLP-vaccinated, bath-challenged survivors had viral RNA in the brain, suggesting a reduced viral entry and/or replication in these fish. As when studying vaccines against diseases, where it is hard to induce disease experimentally, reduced pathogen occurrence may be considered as a proxy of protection [38,39]. The VLP-vaccinated fish all developed a humoral response detectable in ELISA, and the antibodies showed neutralizing effect with titer >640 in a high proportion of the sampled fish at all timepoints (from 3 to almost 9 months pv, Figure 3). The fish receiving the commercial vaccine had lower titers, and in the late T2 challenge, the fish given that vaccine were not protected (Table 1). At that time, only a very low proportion of those fish had neutralizing antibodies (30%), all with low titers (160) (Figure 3). This supported previous findings of an average neutralizing titer above 200 correlating with protection against VNN [26,40]. The antigen concentration and RGNNV strain in the commercial vaccine were unknown to us and may have affected the obtained results. While vaccine efficacies therefore cannot be directly compared, the different results obtained for the two vaccines were still considered useful for understanding mechanisms behind the protective effect of the VLP vaccine reported here.

The antibody status in survivors after experimental challenge is important in a field setting, where it is likely that the survivors from a disease outbreak will encounter the virus again later. We found that the surviving fish from all groups had the same level or a higher level of antibodies than before challenge, both in ELISA and virus neutralization (Figure 6). Only few studies have investigated the level of antibodies in convalescent fish after NNV exposure, and a lack of neutralizing antibodies in previously vaccinated or exposed fish was reported in two studies [26,41]. In another study of serum from convalescent humpback grouper (*Cromileptes altivelis*), which were naïve prior to challenge, neutralizing antibodies were detected 12 days after challenge [15]. We here found that vaccinated and naïve fish surviving bath or injection challenge had neutralizing antibodies at a similar or higher level compared to nonchallenged fish. This indicated that the infection had a boosting effect on the immune response rather than just consuming the produced antibodies, as we have previously discussed [26]. 

As discussed above, previous findings as well as the results reported here suggested a correlation between the antibody response to vaccination and protection. We were therefore surprised to observe that passively immunized recipients with virus-neutralizing titers above 320 in their serum were not protected against the viral disease (Figure 10). However, the results support our earlier findings of lack of correlation between neutralizing antibody levels in serum and survival at the individual level [26]. Whether this reflected that cellular immune mechanisms alone, e.g., cytotoxic T-cells; combined cellular and humoral mechanisms, such as antibody mediated NK-cell killing of virus-infected cells; or the more recently recognized trained innate mechanisms [42] were involved in the long-term immunity induced by the VLP vaccine will require further functional studies. It may be speculated that the neurotrophic nature of NNV might allow the virus infection to propagate to the brain via distal neurons without encountering neutralizing antibodies. The recipients of COM-serum had a slightly increased survival in one of the replicate tanks, suggesting a qualitatively different antibody profile in this group, but because of the tank variability, the experiment should be repeated before such a conclusion can be drawn.

In our studies of the vaccine-induced immune response in gene expression levels, principal component analysis revealed a unique response profile in the VLP-vaccinated fish in all examined immune tissues at day 7 pv that was particularly due to upregulation of IFN-pathway-related genes such as *mx, isg12*, and *ifih1* as well as *mhcI* and *igm*. At day 21 pv, this pattern was still evident in the liver (Figure 7). Mx is known for its antiviral activity in mammals and fish, and specifically for its counteractive effect on the replication of betanodavirus [43,44]. However, while it cannot be excluded that innate defense mechanisms may have contributed to the protection seen here at 3 and 7.5 months post-vaccination, we considered it more likely to be due to adaptive mechanisms. A significant upregulation of adaptive mechanisms was also detected in VLP-vaccinated fish on days 7 and 21, with a 2.5–4.5-fold significant increase in *mhcI*, *mhcII*, *cd4*, *igm* and *igt* in the liver and/or HK (Figure 9). Upregulation of adaptive mechanisms seemed to be slower in the fish receiving the commercial vaccine, in which the strong initial upregulation of *il6* already at day 1 followed by *tnfa* and *tfa* at day 7 possibly reflected the inflammatory response triggered by this oil-adjuvanted vaccine. First on day 21, *cd4*, *igm*, and *igt* were significantly upregulated in this group (in the HK, liver, and/or spleen) (Figure 9).

From mammals, it is known that VLPs are very immunogenic and potent inducers of innate immune mechanisms paving the way for subsequent activation of adaptive responses, and as discussed by Mohsen et al. One explanation for this is the size and the surface geometry of the VLPs [22]. The size of VLPs promotes uptake by phagocytic cells, and the highly repetitive surface structure promotes opsonization by natural IgM and complement C1q, both having opsonin effect and mediating uptake in antigen presenting cells [22]. In teleost fish, the response to VLPs has not been characterized in similar detail, but with IgM as the dominating immunoglobulin class in serum, it may be assumed that opsonization and phagocytosis of VLPs efficiently take place. Also, the repetitive structure of VLPs leads to crosslinking of B-cell receptors and thereby potentially leads to T-cell-independent B-cell activation [22]. Furthermore, fish B-cells have phagocytic activity and presumably also contribute to antigen presentation and thereby immune activation [45]. Our findings of early *igm* and *igt* upregulation might thus reflect involvement of B-cells in the immune response to the VLP vaccine.

The apparent ability of the VLPs to trigger IFN-related immune mechanisms has also been reported in mammals, where recognition by pattern recognition receptors (PRRs) has been linked to both IFN response and uptake in antigen-presenting cells, with subsequent presentation of VLP-derived peptides by MHC I and II cell surface molecules. VLP peptides presented on MHC I, together with production of IL-12, lead to activation of cytotoxic T-cells in mammals [46]. We found *mhcI* upregulation but no increased expression of *cd8b* nor *il12* on the timepoints we examined, and further time-course studies are needed to clarify this. While VLPs in general may be considered as potent PAMPs (pathogen-associated molecular patterns) [22,46], how and by which PRRs they are recognized probably depend on the actual VLP and the host cells used for expression of the viral proteins forming the VLP. Nucleic acids or other components in the VLP-producing host cells may thus be packed into the VLPs in the absence of viral genomes and contribute to recognition by intracellular PRRs [22]. It remains to be examined whether Pichia components packed into or otherwise present in the VLP preparations used in our studies may have contributed to the observed immune response profiles. Two previous studies investigated the immune gene response to NNV VLP vaccination in orange spotted grouper (*Ep. coioides*) [25,47]. As reported here, genes directly related to the IFN pathway and interferon-stimulated genes such as *mx*, *tlr*, and *ifn* were upregulated after vaccination by IM injection. Lin et al. [47] investigated several TLRs (*tlr3*, *tlr9*, *tlr22*) in the spleen, HK, and liver every 4 h from 0 to 72 h after vaccination. They found high variability in expression, with, e.g., the highest expression of *tlr3* and *tlr9* in the liver 24 h after vaccination (~2.8- and ~2-fold, respectively), but the highest expression of *tlr22* at 48 h after vaccination (~1.4-fold) in this organ. How VLPs were expected to upregulate these TLRs, sensing nucleic acid PAMPS directly or indirectly, was not addressed. Here, we accordingly failed to detect any upregulation of the *ifih1* (also known as *mda5*) gene, representing an important PRR activated by viral RNA.

Altogether, our gene expression studies supported earlier observations of VLPs activating both early IFN-related innate immune mechanisms and adaptive arms of immunity. A more comprehensive transcriptomic analysis will be needed to dissect and understand the VLP-induced immune response in further detail, but it may be assumed that the robust and long-lasting response elicited by the VLP vaccine even without adjuvant reflects its ability to activate both innate and adaptive arms in a balanced way. Similar observations have earlier been reported for efficient DNA vaccines against rhabdoviruses in salmonids [48]. Based on our vaccination trials presented here, the VLP-based vaccine produced in Pichia could be administered to fish already at 5 g without safety issues, and the vaccine provided significant long-term protection against disease following experimental challenge, even without formulation with adjuvant. Although the vaccination did not result in sterile immunity in all survivors, fewer VLP-vaccinated survivors had detectable virus in the brain, the target organ of the infection, than mock-vaccinated survivors. Other immune mechanisms than antibodies probably contributed to the long-lasting protection, while a strong and long-lasting antibody response, both in terms of specifically binding activity in ELISA and virus neutralization test, correlated with protective immunity and may be useful for evaluating the immune status of farmed fish stocks.

## 4. Materials and Methods

### 4.1. Virus-like Particles

RGNNV VLPs were produced as previously described [26], although another production batch was used. To confirm the presence of intact VLPs in this batch, these were visualized by transmission electron microscopy, and similar molecular size and antigenicity as the native virus was determined in SDS–PAGE and WB, as previously described [26] (Figure S1). In addition, a WB with ultracentrifuged virus and purified VLP was evaluated with sea bass sera (Figure 2) as detection antibody with the concentrations described in Figure 2. This WB was developed with enhanced chemiluminescent (Pierce ECL, Thermo Fisher Scientific, Waltham, MA, USA) and captured in a chemiluminescent reader (Syngene GeneGnome, Thermo Fisher Scientific, Waltham, MA, USA) in a series of images 30 s apart.

### 4.2. Hyperimmunization of Sea Bass

For the production of sera with high titers of specific and neutralizing antibodies for passive immunization and for reference serum used in ELISA and virus neutralization, larger sea bass (110 g, 15 fish pr. group) were hyperimmunized with 80 µg VLP/fish, commercial vaccine (COM, Alpha Ject micro 1 Noda (Pharmaq A/S, Overhalla, Norway), recommended dose), or mock vaccinated with sterile PBS three times IP (with 3 and 5 weeks between the vaccinations). Blood was harvested from the tail vein three weeks after the last vaccination. After 2 h at room temperature, serum was retrieved by centrifugation at 5000× *g* for 10 min. A working pool of sera for virus neutralization and ELISA was made by mixing an equal amount of serum from all fish receiving the same vaccine (Table 6). After testing all individual sera in ELISA, another pool was made for passive immunization using only the fish with the highest amounts of antibodies (data not shown).

### 4.3. Vaccination

European sea bass (5 ± 2 g) from a commercial breeder (“Ferme Marine du Soleil”, Balaruc-les-Baines, France) (a cross of Atlantic and Western Mediterranean populations) were randomly assigned to three groups and vaccinated intraperitoneally (IP) with 50 µL of either purified VLP (40 µg/fish), commercial vaccine (COM), or sterile PBS as a mock vaccine (*n* = 350/group) with a 29G needle (Table 7). The groups were tagged with fluorescent elastomer tagging (Visible implant elastomer (VIE) tag, Northwest Marine Technology, Anacortes, WA, USA) on the dorsum for identification and equally divided and mixed into two 180 L aerated saltwater tanks (15‰ salinity, 19 °C). The water was flowing in constantly at a temperature of 12 °C, and each tank/bowl was fitted with individual heaters and thermometers to achieve the desired temperature. Fish were fed a commercial feed (2% pr fish body weight) with a feeding machine releasing the feed over a period of 8 h. The fish were kept at a 12 h light and 12 h dark circle. 

After vaccination, the fish were monitored closely for the first 21 days, and any mortality was recorded. Two replicate tanks were monitored. In one of these, the fish (from all groups, VLP, COM, and PBS) started developing wounds laterally on the body on 6 days pv. Fish with wound were euthanized, and swabs were taken from the wounds and head kidney (HK). Growth of *Vibrio splendidus* in pure culture was diagnosed from the wounds on marine agar and confirmed by MALDI-TOF. The samples from the HK were negative. All fish with wounds were euthanized, and treatment with florfincol (8.3 mg/L water) was initiated on day 9 pv and continued once a day for five days. To minimize the differences between the tanks, the healthy tank was also treated. After the treatment, no wounds were visible. A few weeks earlier, some larger sea bass in the stable were diagnosed with wounds from *V. splendidus*, and we suspect that the tank was contaminated from them. Since the wounds occurred in only one of the replicate tanks, and in all groups, we did not suspect that it was caused by the vaccination. However, since the three groups of fish were divided randomly in the two tanks after vaccination, wound development/*V. splendidus* infection must have happened after vaccination. Because of this, the post-vaccination mortality is reported only from the tank without the problems with *V. splendidus*.

### 4.4. Virus and Experimental Challenge

Live RGNNV (strain 283.2009 [49], kindly provided by Dr. Anna Toffan, IZSVe, Italy) was propagated on a 1-day-old monolayer of SSN1 cells [50] at 25 °C as previously described [51]. The cell line was tested free from mycoplasma every 6 months. The fish were challenged with live virus at two timepoints (T1 = 3 and T2 = 7.5 months pv), as specified in Table 8. During the challenges, fish were monitored several times daily, and fish with clinical signs of VNN were euthanized. The experiments were terminated at day 28–32 post-challenge, after at least one week without clinical signs of VNN. A representative number of fish from each group and tank were sampled to confirm the presence of NNV by isolation on cell culture and/or RTqPCR. Surviving fish were euthanized and weighed (in the IM injection challenges). In the T2 challenge, the moribund fish were also weighed. The temperature was kept at 25–26 °C (temperature monitor data are available in Supplementary Figures S2–S5).

### 4.5. Sampling

Five fish per group were euthanized in an overdose of benzocaine on days 1, 7, and 21 pv, and the head kidney, liver, spleen, and brain were sampled in RNA later, stored at +4 °C for 24 h, and thereafter stored at −20 °C until gene expression analysis.

At five time points pv, 5–10 fish per group were euthanized, and blood was sampled from the tail vein with a 1 mL syringe and either a 23 or 25 G single-use needle. The blood was left at room temperature for 1 h or 4 °C over night, followed by centrifugation (5000× *g*, 10 min), and serum was collected and stored at −80 °C until further analysis in ELISA and virus neutralization. Additionally, blood was collected from vaccinated infected survivors (10–12 fish/group/challenge) on days 28–32 post challenge and processed in the same way. From these survivors, the brain was also collected in either RLT buffer (survivors from T1) or L-15 media (1:5) (survivors from T2) for detection of NNV by RTqPCR or isolation on cell culture, respectively. Additionally, a pool of organs (equal amounts of spleen, liver, head kidney, and heart) from the survivors from the T2 challenge were collected in L-15 media (1:5) for virus isolation on cell culture.

A representative number of moribund fish were sampled in each challenge by collecting their brain in L-15 media to reisolate live RGNNV and confirm establishment of the infection.

### 4.6. Detection of NNV by RT-qPCR

RNA was extracted with an Indimag robot (Indical Bioscience, Leipzig, Germany) using bead-based technology. First, the samples were disrupted at 25 Hz for 2 min in a tissue-lyzer (TissueLyser II, Qiagen, Hilden, Germany), spun down and supernatant was collected for RNA extraction with the “pathogen” kit on the Indimag following the manufactures’ protocol.

A multiplex TaqMan one step RT-qPCR targeting both the NNV RNA1 and the housekeeping gene elongation factor 1-alpha (*elf1a*) followed a previously established protocol [51] (Table 9). A dilution series of one sample was included in each run and used to manually set an equal threshold value before exporting the Cq values from MxPro to Microsoft Excel and to calculate the efficiency (Table 9).

### 4.7. Relative Quantification of Virus

The relative amount of RNA1 in the analyzed brains was determined with the 2^–ΔΔCq^ method as described by Barsøe et al. (2021) [51] based on the method originally described by Livak and Schmittgen (2001) [55].

(1) $$∆\mathrm{Cq}={\mathrm{Cq}}_{\mathrm{RNA}1}-{\;\mathrm{Cq}}_{\mathrm{Elf}1\alpha }\;$$

∆∆Cq = ∆Cq_*sample*_ − ∆Cq_*calibrator*_ (2)

where the calibrator was the sample with the least amount of virus (highest ΔCq). The relative quantity (RQ) was then calculated as:

(3) $$\mathrm{RQ}=2^{-∆∆\mathrm{Cq}}$$

Some samples were negative, and they were set as RQ = 0.5 under the assumption that there was virus present, although at lower levels than could be detected. The relative quantities (RQ) were scaled to the group receiving PBS by calculating a scale factor (SF) and multiplying each RQ with this factor:

(4) $$\mathrm{SF}=\frac{1}{\mathrm{mean}\;\left(\mathrm{PBS}\right)}$$

Comparison of means was performed via a *t*-test on data converted into logarithmic scale to obtain linear data. *p*-values less than 0.05 was considered significant, and furthermore, the expression needed to be at least double or half of the expression of the mean expression in the PBS group to be considered biologicall revant.

### 4.8. Isolation of Virus on Cell Culture

Brain or organ pools sampled in L-15 media (1:5) were homogenized in a mortar and centrifuged (4000× *g*, 10 min, 4 °C), and antibiotics (gentamycin 1:50) were added thereafter overnight (4 °C) before inoculation of 150 µL homogenate (undiluted and 1:10) onto a 1-day-old monolayer of SSN1 cells on a 24-well plate (Falcon® primary, Corning, Durham, NC, USA). The plates were incubated for 8 days (25 °C), and if no CPE occurred, a subcultivation was made by transferring a 150 µL mix of the two dilutions per sample (1:1) to a fresh 1-day-old monolayer of SSN1 (24-well plate). The final read for CPE was made after 10 days of incubation. Cell culture supernatant was collected from a representative number of positive wells and analyzed with RTqPCR to confirm the presence of NNV RNA following the protocol described above.

### 4.9. Gene-Expression Analysis Using Microfluidic qPCR

Column-based extraction of RNA with DNAse treatment was performed manually with the RNeasy mini kit (Qiagen, Sweden) according to the manufacturer’s protocol [56]. Duplicate cDNA synthesis and preamplification was conducted as described previously [57] using 500 ng total RNA and 19 cycles of preamplification for all four tissues. qPCR of the preamplified cDNA was performed in 192.24 Dynamic Arrays (Fluidigm), combining 192 preamplified samples with 23 primer sets (Supplementary Table S4) in 4608 individual and simultaneous qPCR reactions as described previously in Barington et al. [58].

### 4.10. Gene-Expression Data Analysis

qPCR data analysis was performed as described previously in Vreman et al. [57] using the following validated reference genes: RPL13a and FAU (kidney, spleen, liver) and ElF1a, RPL13a, and FAU (brain). GeNorm [59] and GeNormFinder [60] were used to identify the most stable reference genes in GenEx6 (MultiD Analyses AB). In samples with no detectable expression (although expression was confirmed in other samples from the same tissue), the relative quantity of mRNA was set to 0.5, which was half the amount of the least measurable amount (which is 1). This value was chosen under the assumption that the mRNA was expressed, although at a level below our detection limit.

### 4.11. ELISA

Serum was diluted in PBS + 5% skimmed milk (1:200 and 1:2000) and applied in duplicates to 96-well ELISA plates (MaxiSorb, In Vitro, Fredensborg, Denmark) coated with 375 ng VLP/well. The plates then incubated at 4 °C over night followed by three washes in PBS-tween20 (0.05%). Rabbit-anti-seabass IgM (1:10,000) ([26], kindly provided by Dr. Anna Toffan, IZSVe, Italy) and swine-anti-rabbit IgG conjugated with HRP (1:1000) (Dako, Glostrup, Denmark) acted as primary and secondary antibodies, respectively, with 1 h (20 °C) incubation and similar wash steps in between. Activation of HRP was initiated with TMB and stopped after 9 min with H_2_SO_4_ when a clear color change was evident. The absorbance was read in an ELISA plate reader (Multiskan FC Microplate Photometer, Thermo Fisher Scientific, Waltham, MA, USA), and the results were the absorbance measured at 450 nm minus the unspecific background measured at 620 nm. Serum from sea bass hyperimmunized with VLP was used as positive reference, and serum from sea bass mock vaccinated with PBS was used as negative reference. Initially, comparison between plates coated with ultracentrifuged live RGNNV (prepared as previously described [26]) and VLP was performed to validate the use of VLP as coating to detect an RGNNV-specific antibody response.

### 4.12. Virus Neutralization

Virus neutralization was performed as previously described [26] with slight modifications. Briefly, serum was twofold diluted from 1:40 to 1:320 and mixed with 100 TCID_50_ RGNNV (strain 283.2009)/well in duplicates, making final serum dilutions of 1:80–1:640. The serum/virus mix was incubated at 4 °C for 18–20 h and inoculated on a <48 h-old monolayer of SSN1 cells the following day. The development of CPE was monitored, and a final read was conducted on day 8. Alternatively, the plates were fixated with 80% acetone (−20 °C) on day 2 post inoculation and stained with polyclonal rabbit-anti-noda (1:3000) ([27], kindly provided by IZSVe) followed by HRP conjugated swine-anti-rabbit secondary antibody (p217, DAKO, Glostrup, Denmark) (1:1000). The reaction was developed with carbazol solution (5 ml sodium acetate, 100 µL carbazol 1%, 100 µL hydrogen peroxide 0.3%), and wells with <10 stained cells were counted as negative (same as “no CPE” in CPE evaluation on day 8). The neutralizing titer was determined as the highest serum dilution with inhibition of CPE (“no CPE”) in both wells. Samples with no inhibition of CPE were denoted as <1:80. In each setup, a control plate with positive and negative reference sera (the same as in ELISA) was included together with a 10-fold serial dilution of the virus, to confirm viral titer of ~100 TCID_50_/well and secure reproducibility.

### 4.13. Passive Immunization and Challenge Experiment

To investigate the protective effect of antibodies in serum from fish immunized with VLP-based vaccine or the commercial vaccine, 270 sea bass (~13 g) were injected intraperitoneally with 100 µL of serum from the hyperimmunized fish (immunized with either VLP-based vaccine, commercial vaccine, or PBS, as described above (Table 10)) (*n* = 90/group). The serum was diluted 1:3 in PBS to achieve a larger volume. The serum was heat treated (45 °C, 30 min) prior to injection to inactivate complement. The groups were equally and randomly distributed into two tanks for challenge (*n* = 40/group/tank), while 10 fish per group were kept in a separate nonchallenged tank with 90 naïve sea bass. These were blood sampled on day 1. The fish were challenged IM with 10^4^ TCID_50/_fish RGNNV strain 283.2009 on day 1 after passive immunization in the same way as described for the vaccinated fish.

### 4.14. Statistical Evaluation

The probability of a fish surviving until the end of the experiment ranged from 0 to 1 (0–100%), and survival was assumed to be binomially distributed. To test whether the probability of surviving was influenced by the different vaccines, a logistic regression (i.e., binomial GLMM) was applied in R (ver. 4.0.3) using the package “glmmTMB” [61,62]. The effect of the replicate tanks was added as a random effect for the experiments with more than two replicate tanks (T1 bath and T1 injection), while it was set as a fixed effect (factor) in the experiments with two replicate tanks (T2 and passive immunization):

logit(p_surv_i_) = α + β_treatment_i_, ε_tank_t_i_ (tank as random effect) (5)

or

logit(p_surv_i_) = α + β_treatment_i_ + β_tank_t_i_ (tank as a factor) (6)

where p_surv_i_ is the probability of surviving for the individual fish with the treatment = treatment_i in the tank = t_i. The tank effect accounted for potential variation among tanks due to random factors such as behavior and temperature. The odds of surviving following vaccination:

(7) $$\mathrm{odds}\left({\mathrm{surv}}_{{\mathrm{treatment}}_{t}}\right)=\frac{p\left({\mathrm{surv}}_{{\mathrm{treatment}}_{t}}\right)}{1-p\left({\mathrm{surv}}_{{\mathrm{treatmnent}}_{t}}\right)}$$

were compared by estimating the odds ratio (OR) between two treatment groups:

(8) $$\mathrm{OR}=\frac{\mathrm{odds}\left({\mathrm{surv}}_{{\mathrm{treatment}}_{t}}\right)}{\mathrm{odds}\left({\mathrm{surv}}_{{\mathrm{treatment}}_{j}}\right)}$$

with the package “emmeans” [63]. Thus, if OR > 1 (and *p* < 0.05), treatment t significantly increased the odds of surviving the experimental challenge compared to treatment j. The opposite held for OR < 1, while OR with *p* > 0.05 indicated that there was no significant difference in the odds of surviving on a 95% test level.

Survival analysis and principal component analysis (PCA) were also performed in R [62]. Survival curves were drawn with the package Survminer [64], and illustrations were made with the package ggplot2 [65]. Data of qPCR results from both gene expression and NNV diagnostics were handled in Microsoft Excel, with which the bar charts and *t*-test comparisons were also made. Likewise, the data handling of ELISA and SN results and the calculation of means, SDs, and proportions were conducted in Microsoft Excel, with which the graphs were also made.

## Figures and Tables

**Table 1:** Survival percentage after experimental challenges.

		Survival Percentage (%)										Bowl ^1^		Fish ^2^		
Replicate		I	II	III	IV	V	VI	VII	VIII	IX	X	Mean	SD	Surv %	SD	RPS ^3^
Time	Group															
T1 Bath	PBS	75.0	85.7	100.0	57.1	71.4	57.1	83.3	100.0	85.7	100.0	81.5	16.3	80.9	4.8	
	VLP	100.0	85.7	100.0	100.0	85.7	71.4	100.0	100.0	100.0	100.0	94.3	10.0	94.3	2.8	70
	COM	87.5	57.1	71.4	85.7	57.1	42.9	75.0	42.9	85.7	100.0	70.5	19.8	70.8	5.4	-
T1 Inj	PBS	71.4	42.9	42.9	42.9	71.4						54.3	12.8	54.3	8.4	
	VLP	100.0	71.4	100.0	100.0	100.0						94.3	12.8	94.3	3.9	88
	COM	100.0	71.4	66.7	28.6	100.0						73.3	29.5	71.0	8.2	37
T2 Inj	PBS	13.6	39.1									26.4	18.0	26.7	6.6	
	VLP	89.3	92.0									90.6	1.9	90.6	4.0	87
	COM	19.2	35.7									27.5	11.7	27.8	6.1	2

Endpoint survival in each replicate tank. The fish were tagged, and the three groups (PBS, VLP, commercial vaccine) were mixed equally in each tank. The endpoint survival was counted on day 32 (T1 bath and T2 inj) or day 22 (T1 inj) after challenge. T1 challenge = 93 days pv (~1800 dd), T2 challenge = 240 days pv (~4500 dd). Temperature data from each challenge is available in Figures S2–S5. ^1^ Mean endpoint survival and SD for all replicate bowls (observation = bowl). ^2^ Mean endpoint survival and SD for pooled bowls (observation = fish). ^3^ RPS = relative percent survival = (1 − (mortality%(vaccine)/mortality%(PBS)))∗100 [28].

**Table 2:** Odds ratios of survival dependent on treatment in the different experimental challenges and significance thereof.

Treatments	T1 Bath			T1 Injection			T2 Injection		
	OR	SE	*p*	OR	SE	p	OR	SE	*p*
VLP-PBS	4.1	2.51	0.0557	**16.5**	**13.79**	**0.0031**	**31.5**	**19.00**	**<0.0001**
VLP-COM	**7.5**	**4.42**	**0.0022**	7.1	5.97	0.059	**30.0**	**17.49**	**<0.0001**
PBS-COM	1.8	0.76	0.3239	0.4	0.24	0.2807	1.0	0.44	0.9932

Estimated odds ratios of survival (ORs) and their standard errors (SEs) from the glmmTMB model using the emmeans function. Significant ORs (*p*-values < 0.05) are marked in bold. Interpretation of OR is explained in methods, Section 4.14.

**Table 3:** Detection of virus or viral RNA from tissues of healthy survivors.

Challenge	Detection Method	Tissue	PBS	VLP	COM
T1 inj	RTqPCR	Brain	100% (6/6)	33% (2/6) *	100% (6/6)
T1 bath	RTqPCR	Brain	80% (8/10)	30% (3/10) *	60% (6/10)
T2 inj	Cell culture	Brain	100% (9/9)	83% (10/12)	100% (11/11)
	Cell culture	Organs ^1^	67% (6/9)	33% (4/12)	73% (8/11)

^1^ One pool per fish with equal amounts of heart, spleen, head kidney, and liver. Tissue for cell-culture isolation of virus was processed as described in methods. Positive samples showed clear cytopathic effect in either primary or subcultivation within 10 days after inoculation at 25 °C incubation. The presence of nervous necrosis virus in the cell culture supernatant was confirmed by RTqPCR in a representative number of wells with CPE. * Proportion significantly different from PBS (*p* < 0.05, Chi–squared test).

**Table 4:** Survival % of passively immunized sea bass challenged with RGNNV.

Donor Serum	Replicate		Tank		Fish	
	Rep. I %	Rep. II %	Average %	SD	Average %	SD
PBS	35.3 (12/34)	53.7 (22/41)	44.5	9.2	45.3 (34/75)	5.7
VLP	32.4 (12/37)	44.4 (16/36)	38.4	6.0	38.4 (28/73)	5.7
COM	48.6 (18/37)	80.0 (28/35)	64.3	15.7	63.9 (46/72)	5.6

Survival percentage of sea bass passively immunized with donor serum from fish hyperimmunized with either phosphate-buffered saline (PBS), virus-like particle vaccine (VLP), or commercial vaccine (COM). The numbers in brackets are the actual numbers leading to the percentages.

**Table 5:** Odds ratio of surviving experimental challenge after receipt of donor serum.

Treatments	OR	SE	*p*-Value
VLP–PBS	0.758	0.245	0.6682
VLP–COM	0.353	0.119	**0.0064**
PBS–COM	0.466	0.155	0.0584

Estimated odds ratios of survival (ORs) and their standard errors (SEs) from the glmmTMB model using the emmeans function. Significant *p*-values are marked in bold. Interpretation of OR is explained in methods, Section 4.14.

**Table 6:** Positive reference serum for virus neutralization and ELISA.

Serum Pool	Neutralizing Titer	ELISA Abs (1:200) (SD)
PBS	<80	0.1 (0.00)
VLP	81.920	2.1 (0.06)
COM	ND	1.1 (0.06)

ND = not determined, SD = standard deviation of duplicate samples.

**Table 7:** Treatments.

Vaccine	Type	Adjuvant	Concentration	Virus Species
VLP	Virus-like particle	none	~800 µg/mL ^1^	RGNNV
Commercial vaccine (COM)	Inactivated virus	mineral oil	unknown	RGNVV
PBS	Phosphate-buffered saline	none	NA	NA

^1^ Determined by Bradford assay; NA = Not applicable.

**Table 8:** Experimental challenges of European sea bass vaccinated with either VLP-based vaccine, commercial vaccine, or PBS.

Days Postvac.	Average Size	Infection Route	Infected Tanks ^1^	Control Tanks ^1^	Fish per Tank	Temperature (°C ± 1) ^2^	Virus Dose
98 (~3 months)	15 g	Bath	10 × 10 L	2 × 10 L	21 (7/group)	25	4 × 10^5^ TCID_50_/_mL_^4^
	15 g	IM ^3^	7 × 10 L	2 × 10 L	21 (7/group)	25	8.5 × 10^2^ TCID_50_/fish ^5^
237 (~7.5 months)	44 g	IM ^3^	2 × 180 L	1 × 180 L	75 (~25/group)	26	10^5^ TCID_50_/fish ^5^

^1^ The groups were equally mixed in each tank. ^2^ The water temperature was 20 °C at the time of challenge, whereafter it was gradually increased to reach the set temperature (25 or 26 °C) at day 3 after challenge. The water flowed in constantly at a temperature of 12 °C, and each tank/bowl was fitted with individual heaters and thermometers to achieve the desired temperature. Temperature recordings are available in Supplementary Figures S2–S5. ^3^ IM = intramuscularly. ^4^ TCID_50_/_mL_ = TCID_50_ per mL of saltwater. TCID_50_ determined as in [52]. The fish were kept in the infected water for 2.5 h, where after the flow was started and gradually changed the water. A water sample was taken before starting the flow to reisolate the virus. Nodavirus was successfully isolated from all bath-infected tanks at titers from 3.2 × 10^2^ to 1.5 × 10^5^ TCID_50_/_mL_. ^5^ TCID_50_/fish = TCID_50_ injected in each fish (volume = 50 µL).

**Table 9:** Primers and probes for multiplex RTqPCR.

Target Gene	5′-3′ Sequence	Reference	nM	Efficiency
*elf1a*	F: GGAGTGAAGCAGCTCATCGTT	[53]	200	98.4–99.4%
	R: GCGGGCCTGGCTGTAAG		300	
	P: [5HEX]AGTCAA[ZEN]CAAGATGGACTCCACTGAGCCC[3 IABkFQ]		50	
RNA1	F: TCCAAGCCGGTCCTAGTCAA	[54]	600	96.1–99.0%
	R: CACGAACGTKCGCATCTCGT		600	
	P: [6FAM]CGATCGATC[ZEN]AGCACCTSGTC[3IABkFQ]		400	

*elf1a* = elongation factor 1-alpha, RNA1 = nervous necrosis virus RNA1, F = forward primer, R = reverse primer, P = probe.

**Table 10:** Donor serum for passive immunization.

Donor Serum Pool	Neutralizing Titer	ELISA Abs (1:200)
PBS	<80	0.12 (±0.02)
VLP	>10.240	2.10 (±0.02)
COM	1:1280	1.03 (±0.02)

## Data Availability

Data is available from the authors upon request.

## Supplementary Materials

The following are available online at https://www.mdpi.com/article/10.3390/pathogens10111477/s1: Figure S1: SDS–PAGE and Western blot; Figures S2–S5: Water temperature curves from challenge experiments; Video S1: Video showing clinical signs of VNN from day 8, vaccinated European sea bass bath challenge with RGNNV. Both “typical” clinical signs (the large fish swirling, mock-vaccinated control) and characteristic clinical signs seen in the fish receiving the commercial vaccine (the smaller, very dark fish with hemorrhagic nasal area); Table S1: Record of mortality and weight after vaccination and challenge; Tables S2–S4: Excel file with tables relating to gene-expression data.

## References

[1] Vandeputte M., Gagnaire P.A., Allal F. (2019). The European sea bass: A key marine fish model in the wild and in aquaculture. Anim. Genet..

[2] Le Breton A., Grisez L., Sweetman J., Ollevier F. (1997). Viral nervous necrosis (VNN) associated with mass mortalities in cage-reared sea bass, *Dicentrarchus labrax* (L.). J. Fish Dis..

[3] Chérif N., Thiéry R., Castric J., Biacchesi S., Brémont M., Thabti F., Limem L., Hammami S. (2009). Viral encephalopathy and retinopathy of Dicentrarchus labrax and Sparus aurata farmed in Tunisia. Vet. Res. Commun..

[4] Muniesa A., Basurco B., Aguilera C., Furones D., Reverté C., Sanjuan-Vilaplana A., Jansen M.D., Brun E., Tavornpanich S. (2020). Mapping the knowledge of the main diseases affecting sea bass and sea bream in Mediterranean. Transbound. Emerg. Dis..

[5] Vendramin N., Toffan A., Mancin M., Cappellozza E., Panzarin V., Bovo G., Cattoli G., Capua I., Terregino C. (2014). Comparative pathogenicity study of ten different betanodavirus strains in experimentally infected European sea bass, *Dicentrarchus labrax* (L.). J. Fish Dis..

[6] Vendramin N., Zrncic S., Padrós F., Oraic D., Le Breton A., Zarza C., Olesen N.J. (2016). Fish health in Mediterranean Aquaculture, past mistakes and future challenges. Bull. Eur. Assoc. Fish Pathol..

[7] Sahul Hameed A.S., Ninawe A.S., Nakai T., Chi S.C., Johnson K.L. (2019). ICTV Virus Taxonomy Profile: Nodaviridae. J. Gen. Virol..

[8] Chen N.C., Yoshimura M., Guan H.H., Wang T.Y., Misumi Y., Lin C.C., Chuankhayan P., Nakagawa A., Chan S.I., Tsukihara T. (2015). Crystal Structures of a Piscine Betanodavirus: Mechanisms of Capsid Assembly and Viral Infection. PLoS Pathog..

[9] Bandín I., Souto S. (2020). Betanodavirus and VER disease: A 30-year research review. Pathogens.

[10] Buonocore F., Nuñez-Ortiz N., Picchietti S., Randelli E., Stocchi V., Guerra L., Toffan A., Pascoli F., Fausto A.M., Mazzini M. (2019). Vaccination and immune responses of European sea bass (*Dicentrarchus labrax* L.) against betanodavirus. Fish Shellfish Immunol..

[11] Hipra Icthiovac VNN. https://www.hipra.com/portal/fr/hipra/animalhealth/products/detail-global/icthiovac-vnn.

[12] Pharmaq ALPHA JECT micro® 1 Noda. https://www.pharmaq.no/sfiles/2/54/9/file/product-info_alpha-jet_micro-1-noda_english_2018-9.pdf.

[13] Sommerset I., Skern R., Biering E., Bleie H., Fiksdal I.U., Grove S., Nerland A.H. (2005). Protection against Atlantic halibut nodavirus in turbot is induced by recombinant capsid protein vaccination but not following DNA vaccination. Fish Shellfish Immunol..

[14] Thiéry R., Cozien J., Cabon J., Lamour F., Baud M., Schneemann A. (2006). Induction of a protective immune response against viral nervous necrosis in the European sea bass *Dicentrarchus labrax* by using betanodavirus virus-like particles. J. Virol..

[15] Yuasa K., Koesharyani I., Roza D., Mori K., Katata M., Nakai T. (2002). Immune response of humpback grouper, Cromileptes altivelis (Valenciennes) injected with the recombinant coat protein of betanodavirus. J. Fish Dis..

[16] Nuñez-Ortiz N., Pascoli F., Picchietti S., Buonocore F., Bernini C., Toson M., Scapigliati G., Toffan A. (2016). A formalin-inactivated immunogen against viral encephalopathy and retinopathy (VER) disease in European sea bass (*Dicentrarchus labrax*): Immunological and protection effects. Vet. Res..

[17] Húsgaro S., Grotmol S., Hjeltnes B.K., Rødseth O.M., Biering E. (2001). Immune response to a recombinant capsid protein of striped jack nervous necrosis virus (SJNNV) in turbot *Scophthalmus maximus* and Atlantic halibut *Hippoglossus hippoglossus*, and evaluation of a vaccine against SJNNV. Dis. Aquat. Organ..

[18] Kim H.J., Kim H.J. (2017). Yeast as an expression system for producing virus-like particles: What factors do we need to consider?. Lett. Appl. Microbiol..

[19] Nooraei S., Bahrulolum H., Hoseini Z.S., Katalani C., Hajizade A., Easton A.J., Ahmadian G. (2021). Virus-like particles: Preparation, immunogenicity and their roles as nanovaccines and drug nanocarriers. J. Nanobiotechnol..

[20] Jeong H., Seong B.L. (2017). Exploiting virus-like particles as innovative vaccines against emerging viral infections. J. Microbiol..

[21] Fougeroux C., Goksøyr L., Idorn M., Soroka V., Myeni S.K., Dagil R., Janitzek C.M., Søgaard M., Aves K.L., Horsted E.W. (2021). Capsid-like particles decorated with the SARS-CoV-2 receptor-binding domain elicit strong virus neutralization activity. Nat. Commun..

[22] Mohsen M.O., Gomes A.C., Vogel M., Bachmann M.F. (2018). Interaction of viral capsid-derived virus-like particles (VLPs) with the innate immune system. Vaccines.

[23] Lu M.W., Liu W., Lin C.S. (2003). Infection competition against grouper nervous necrosis virus by virus-like particles produced in Escherichia coli. J. Gen. Virol..

[24] Liu W., Hsu C.H., Chang C.Y., Chen H.H., Lin C.S. (2006). Immune response against grouper nervous necrosis virus by vaccination of virus-like particles. Vaccine.

[25] Lai Y.X., Jin B.L., Xu Y., Huang L.J., Huang R.Q., Zhang Y., Kwang J., He J.G., Xie J.F. (2014). Immune responses of orange-spotted grouper, Epinephelus coioides, against virus-like particles of betanodavirus produced in Escherichia coli. Vet. Immunol. Immunopathol..

[26] Barsøe S., Toffan A., Pascoli F., Stratmann A., Pretto T., Marsella A., Er M. (2021). Long-term protection and serologic response of European sea bass vaccinated with a betanodavirus Virus-like Particle produced in *Pichia pastoris*. Vaccines.

[27] Panzarin V., Toffan A., Abbadi M., Buratin A., Mancin M., Braaen S., Olsen C.M., Bargelloni L., Rimstad E., Cattoli G. (2016). Molecular basis for antigenic diversity of genus Betanodavirus. PLoS ONE.

[28] Amend D.F. (1981). Potency Testing of Fish Vaccines. Fish Biol. Serodiagn. Vaccines.

[29] Cecchini S., Saroglia M. (2002). Antibody response in sea bass (*Dicentrarchus Labrax* L.) in relation to water temperature and oxygenation. Aquac. Res..

[30] Gonzalez-Silvera D., Guardiola F.A., Espinosa C., Chaves-Pozo E., Esteban M.Á., Cuesta A. (2019). Recombinant nodavirus vaccine produced in bacteria and administered without purification elicits humoral immunity and protects European sea bass against infection. Fish Shellfish Immunol..

[31] Marsian J., Hurdiss D.L., Ranson N.A., Ritala A., Paley R., Cano I., Lomonossoff G.P. (2019). Plant-Made Nervous Necrosis Virus-Like Particles Protect Fish Against Disease. Front. Plant Sci..

[32] Wi G.R., Hwang J.Y., Kwon M.G., Kim H.J., Kang H.A., Kim H.J. (2015). Protective immunity against nervous necrosis virus in convict grouper *Epinephelus septemfasciatus* following vaccination with virus-like particles produced in yeast *Saccharomyces cerevisiae*. Vet. Microbiol..

[33] Cho S.Y., Kim H.J., Lan N.T., Han H.J., Lee D.C., Hwang J.Y., Kwon M.G., Kang B.K., Han S.Y., Moon H. (2017). Oral vaccination through voluntary consumption of the convict grouper Epinephelus septemfasciatus with yeast producing the capsid protein of red-spotted grouper nervous necrosis virus. Vet. Microbiol..

[34] Midtlyng P.J., Lillehaug A. (1995). Growth of Atlantic salmon Salmo salar after intraperitoneal administration of vaccines containing adjuvants. Atlantic.

[35] Midtlyng P.J., Reitan L.J., Speilberg L. (1996). Experimental studies on the efficacy and side-effects of intraperitoneal vaccination of Atlantic salmon (*Salmo salar* L.) against furunculosis. Fish Shellfish Immunol..

[36] Lampou E., Dovas C., Margaroni M., Chasalevris T., Pappas I.S., Dotsika E., Karagouni E., Athanassopoulou F., Katsaras D., Bitchava K. (2020). Investigation of routes of entry and dispersal pattern of RGNNV in tissues of European sea bass, *Dicentrarchus labrax*. J. Fish Dis..

[37] Toffan A., Panzarin V., Toson M., Cecchettin K., Pascoli F. (2016). Water temperature affects pathogenicity of different betanodavirus genotypes in experimentally challenged Dicentrarchus labrax. Dis. Aquat. Organ..

[38] Wessel Ø., Haugland Ø., Rode M., Fredriksen B.N., Dahle M.K., Rimstad E. (2018). Inactivated Piscine orthoreovirus vaccine protects against heart and skeletal muscle inflammation in Atlantic salmon. J. Fish Dis..

[39] Malik M.S., Teige L.H., Braaen S., Olsen A.B., Nordberg M., Amundsen M.M., Dhamotharan K., Svenning S., Edholm E.S., Takano T. (2021). Piscine Orthoreovirus (PRV)-3, but Not PRV-2, Cross-Protects against PRV-1 and Heart and Skeletal Muscle Inflammation in Atlantic Salmon. Vaccines.

[40] Yamashita H., Mori K., Kuroda A., Nakai T. (2009). Neutralizing antibody levels for protection against betanodavirus infection in sevenband grouper, epinephelus septemfasciatus (Thunberg), immunized with an inactivated virus vaccine. J. Fish Dis..

[41] Gye H.J., Oh M.J., Nishizawa T. (2018). Lack of nervous necrosis virus (NNV) neutralizing antibodies in convalescent sevenband grouper Hyporthodus septemfasciatus after NNV infection. Vaccine.

[42] Petit J., Wiegertjes G.F. (2016). Long-lived effects of administering β-glucans: Indications for trained immunity in fish. Dev. Comp. Immunol..

[43] Wu Y.C., Lu Y.F., Chi S.C. (2010). Anti-viral mechanism of barramundi Mx against betanodavirus involves the inhibition of viral RNA synthesis through the interference of RdRp. Fish Shellfish Immunol..

[44] Chen Y.M., Su Y.L., Shie P.S., Huang S.L., Yang H.L., Chen T.Y. (2008). Grouper Mx confers resistance to nodavirus and interacts with coat protein. Dev. Comp. Immunol..

[45] Mutoloki S., Jørgensen J.B., Evensen Ø., Gudding R., Lillehaug A., Evensen Ø. (2014). The Adaptive Immune Response in Fish. Fish Vaccination.

[46] Zepeda-Cervantes J., Ramírez-Jarquín J.O., Vaca L. (2020). Interaction between Virus-like Particles (VLPs) and Pattern Recognition Receptors (PRRs) from Dendritic Cells (DCs): Toward Better Engineering of VLPs. Front. Immunol..

[47] Lin K., Zhu Z., Ge H., Zheng L., Huang Z., Wu S. (2016). Immunity to nervous necrosis virus infections of orange-spotted grouper (*Epinephelus coioides*) by vaccination with virus-like particles. Fish Shellfish Immunol..

[48] Collins C., Lorenzen N., Collet B. (2019). DNA vaccination for finfish aquaculture. Fish Shellfish Immunol..

[49] Panzarin V., Fusaro A., Monne I., Cappellozza E., Patarnello P., Bovo G., Capua I., Holmes E.C., Cattoli G. (2012). Molecular epidemiology and evolutionary dynamics of betanodavirus in southern Europe. Infect. Genet. Evol..

[50] Frerichs G.N., Rodger H.D., Peric Z. (1996). Cell culture isolation of piscine neuropahy nodavirus from juvenile sea bass, Dicentrarchus labrax. J. Gen. Virol..

[51] Barsøe S., Allal F., Vergnet A., Vandeputte M., Olesen N.J., Schmidt J.G., Larsen C.A., Cuenca A., Vendramin N. (2021). Different survival of three populations of European sea bass (*Dicentrarchus labrax*) following challenge with two variants of nervous necrosis virus (NNV). Aquac. Rep..

[52] Reed L.J., Muench H. (1938). A Simple Method of Estimating Fifty Percent Endpoints. Am. J. Epidemiol..

[53] Rocha A., Zanuy S., Carrillo M., Gómez A. (2009). Seasonal changes in gonadal expression of gonadotropin receptors, steroidogenic acute regulatory protein and steroidogenic enzymes in the European sea bass. Gen. Comp. Endocrinol..

[54] Baud M., Cabon J., Salomoni A., Toffan A., Panzarin V., Bigarré L. (2015). First generic one step real-time Taqman RT-PCR targeting the RNA1 of betanodaviruses. J. Virol. Methods.

[55] Livak K.J., Schmittgen T.D. (2001). Analysis of relative gene expression data using real-time quantitative PCR and the 2−ΔΔCT method. Methods.

[56] (2019). QIAGEN RNeasy Mini Handbook. RNeasy® Mini Handbook.

[57] Vreman S., Rebel J.M.J., McCaffrey J., Ledl K., Arkhipova K., Collins D., McDaid D., Savelkoul H.F.J., Skovgaard K., Moore A.C. (2021). Early immune responses in skin and lymph node after skin delivery of Toll-like receptor agonists in neonatal and adult pigs. Vaccine.

[58] Barington K., Jensen H.E., Skovgaard K. (2018). Forensic age determination of human inflicted porcine bruises inflicted within 10 h prior to slaughter by application of gene expression signatures. Res. Vet. Sci..

[59] Vandesompele J., De Preter K., Pattyn F., Poppe B., Van Roy N., De Paepe A., Speleman F. (2002). Accurate normalization of real-time quantitative RT-PCR data by geometric averaging of multiple internal control genes. Genome Biol..

[60] Andersen C.L., Jensen J.L., Ørntoft T.F. (2004). Normalization of real-time quantitative reverse transcription-PCR data: A model-based variance estimation approach to identify genes suited for normalization, applied to bladder and colon cancer data sets. Cancer Res..

[61] Brooks M.E., Kristensen K., van Benthem K.J., Magnusson A., Berg C.W., Nielsen A., Skaug H.J., Maechler M., Bolker B.M. (2017). glmmTMB Balances Speed and Flexibility Among Packages for Zero-inflated Generalized Linear Mixed Modeling. R J..

[62] R Core Team R: A Language and Environment for Statistical Computing. www.R-project.org.

[63] Lenth R. V emmeans: Estimated Marginal Means, aka Least-Squares. Means. https://cran.r-project.org/package=emmeans.

[64] Kassambara A., Kosinski M., Biecek P. Survminer: Drawing Survival Curves Using “ggplot2”. https://cran.r-project.org/package=survminer.

[65] Wickham H. (2016). ggplot2: Elegant Graphics for Data Analysis.

