# Supplementary material for: Nervous Necrosis Virus-like Particle (VLP) Vaccine Stimulates European Sea Bass Innate and Adaptive Immune Responses and Induces Long-Term Protection against Disease
Source: Pathogens. 2021 Nov 12;10(11):1477. doi: 10.3390/pathogens10111477 (PMC8623669; doi:10.3390/pathogens10111477)
Supplement: Supplementary file 1 [file pathogens-10-01477-s001.zip › Fig S1.pdf]

Supplementary figure to Barsøe et al 2021 “Nervous Necrosis Virus-like Particles (VLP) vaccine stimulates European Sea Bass Innate and Adaptive Immune Responses and induces long-term protection against disease”

Figure S1: SDS-Page gel and western blot of VLP and RGNNV

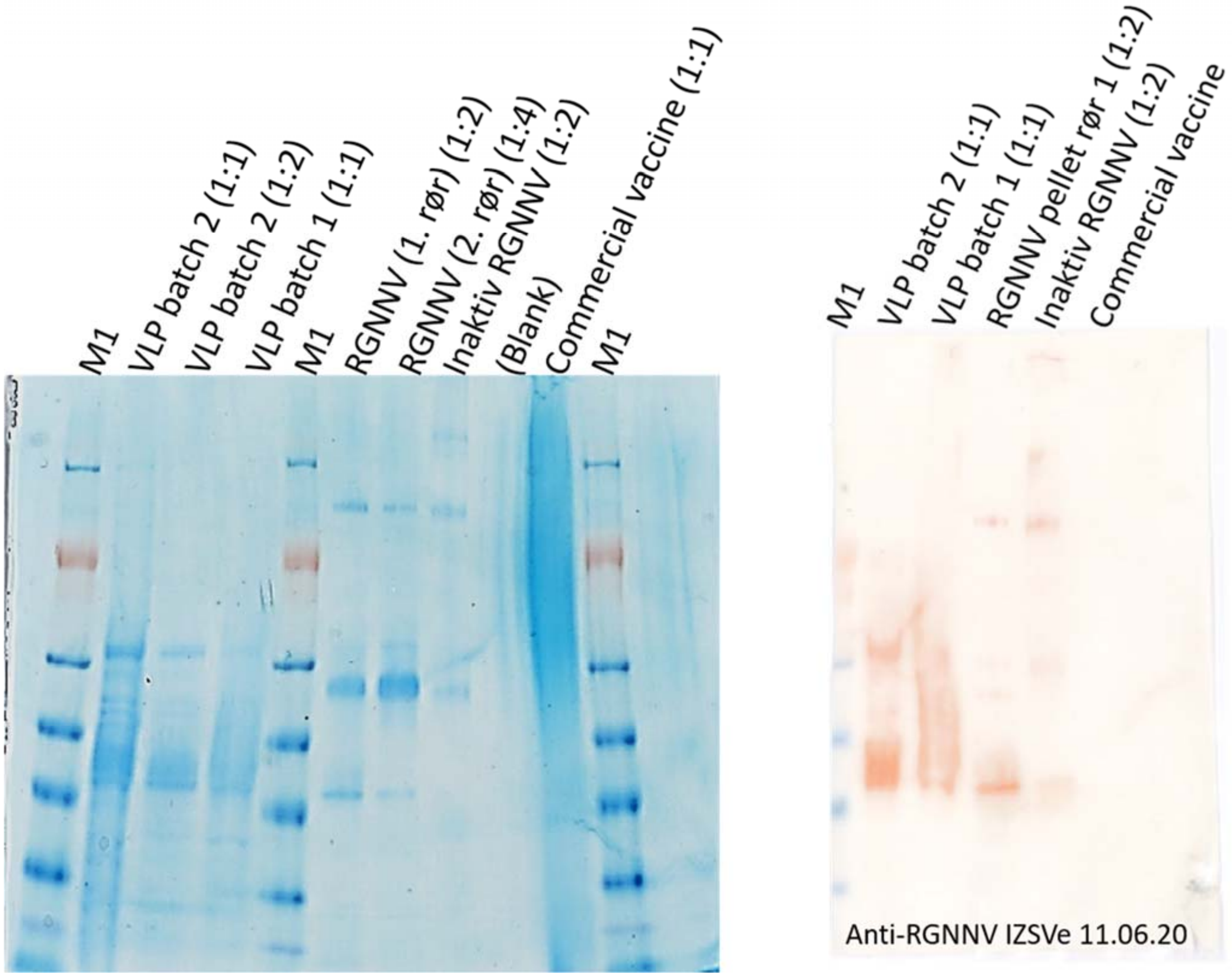

VLP batch 1 is the batch used in Barsøe et al. (2021). VLP batch 2 is the batch used in this manuscript. RGNNV is ultracentrifuged RGNNV as described in Barsøe et al. (2021). “Inactive RGNNV” and “commercial vaccine” is not included in this manuscript.

Supplementary figure to Barsøe et al 2021 “Nervous Necrosis Virus-like Particles (VLP) vaccine stimulates European Sea Bass Innate and Adaptive Immune Responses and induces long-term protection against disease”

M1 = Marker (NOVEX, SeeBlue Plus2, Pre-Stained Standards**,** 250–4 kDa**,** 10 bands). Please not that the molecular weight cannot be interpreted as it is a pre-stained ladder. (Picture from the manufacturers webpage: https://www.thermofisher.com/order/catalog/product/LC5925#/LC5925)

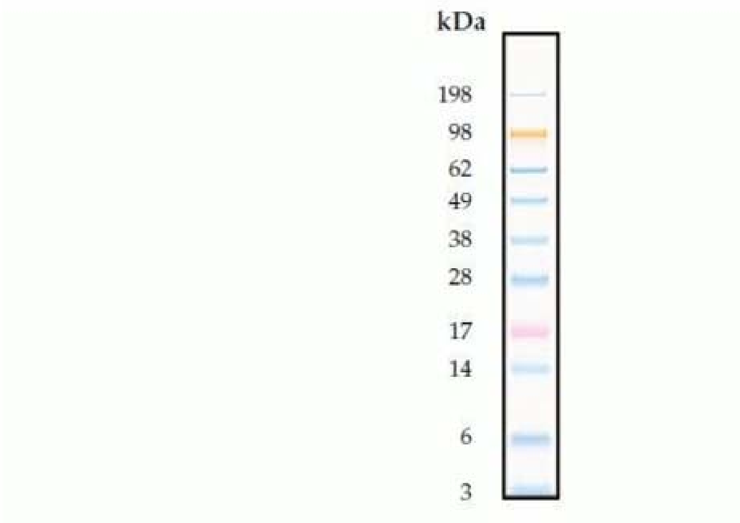

SeeBlue® Plus2 Pre-Stained Standard.
Apparent molecular weights of the SeeBlue® Pre-Stained Standard on a NuPAGE® Novex® 4-12% Bis-Tris Gel with MES.
