# Supplementary material for: Nervous Necrosis Virus-like Particle (VLP) Vaccine Stimulates European Sea Bass Innate and Adaptive Immune Responses and Induces Long-Term Protection against Disease
Source: Pathogens. 2021 Nov 12;10(11):1477. doi: 10.3390/pathogens10111477 (PMC8623669; doi:10.3390/pathogens10111477)
Supplement: Supplementary file 1 [file pathogens-10-01477-s001.zip › Figures S2-5 (temperature curves).pdf]

Supplementary figures to Barsøe et al 2021 “Nervous Necrosis Virus-like Particles (VLP) vaccine stimulates European Sea Bass Innate and Adaptive Immune Responses and induces long-term protection against disease”

## Figures S2-5 – Water temperature curves from challenge experiments

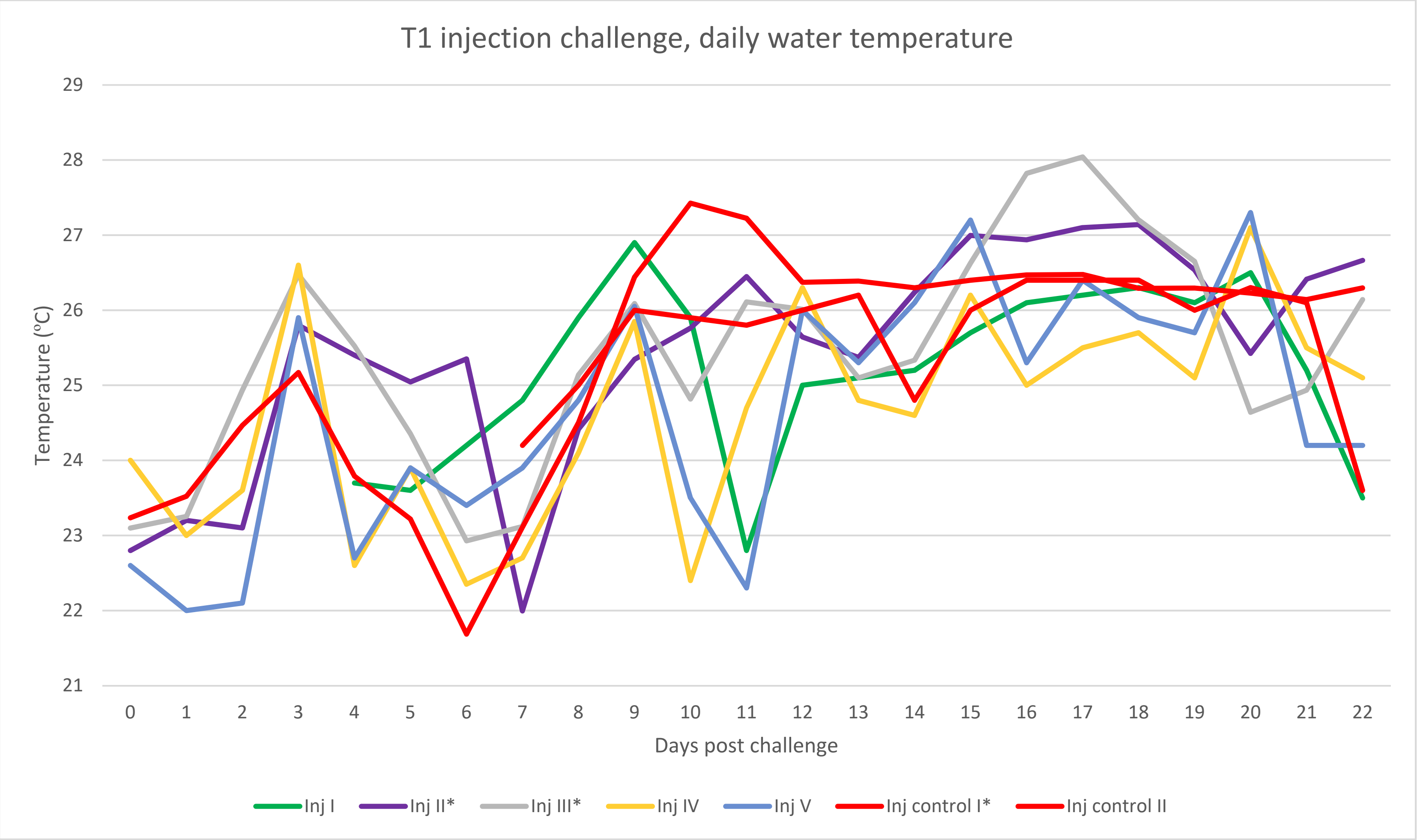


*Figure S2: Temperature curve for replicates in T1-challenge injection. The replicates marked with * was monitored with a temperature logger, logging every 30 min. The noted temperature for these is the average temperature pr. day. The replicate numbers refer to the replicates in table 2.*

Supplementary figures to Barsøe et al 2021 “Nervous Necrosis Virus-like Particles (VLP) vaccine stimulates European Sea Bass Innate and Adaptive Immune Responses and induces long-term protection against disease”

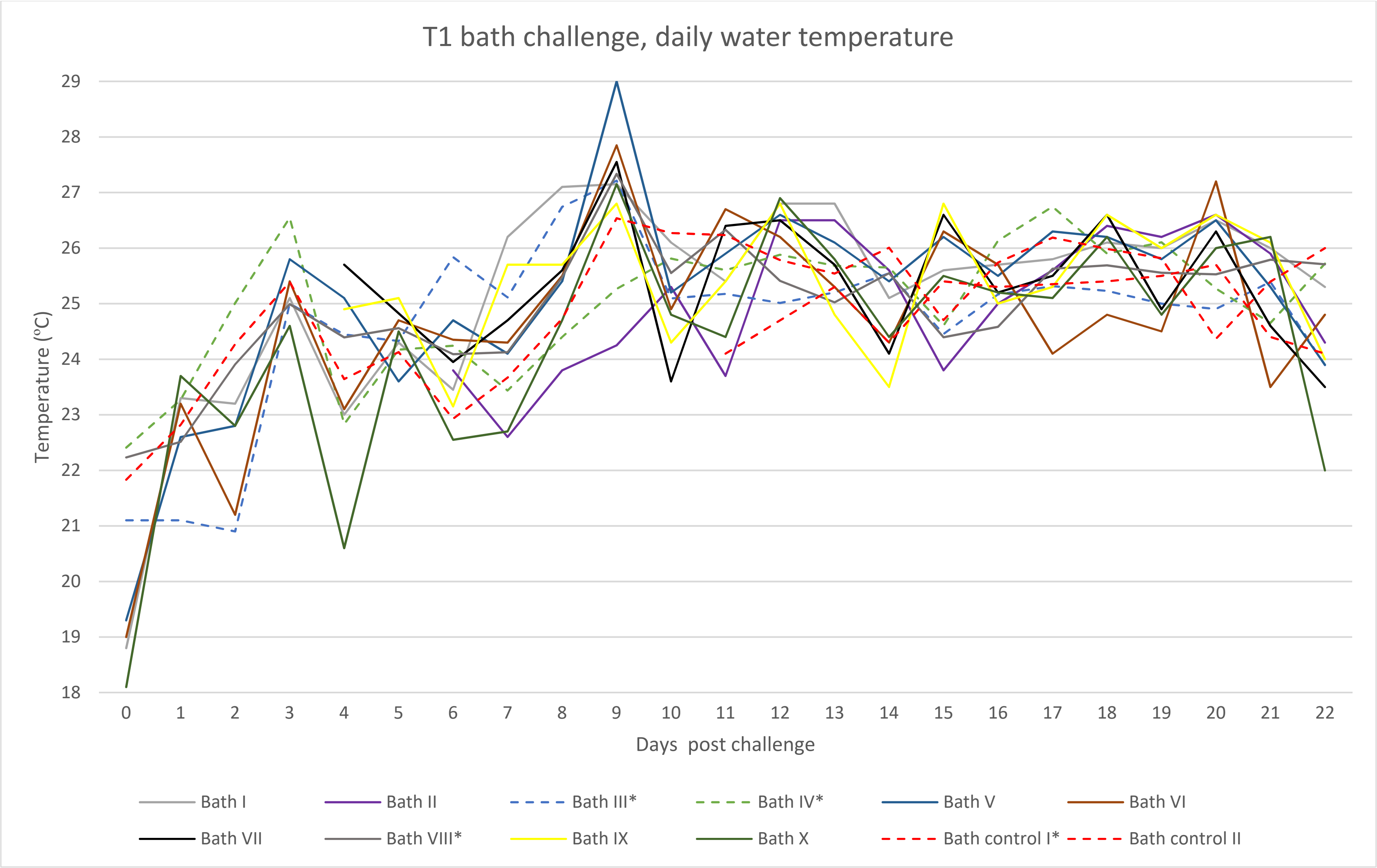


*Figure S3: Temperature curve for replicates in T1-challenge bath. The replicates marked with * was monitored with a temperature logger, logging every 30 min. The noted temperature for these is the average temperature pr. day. The replicate numbers refer to the replicates in table 2.*

Supplementary figures to Barsøe et al 2021 “Nervous Necrosis Virus-like Particles (VLP) vaccine stimulates European Sea Bass Innate and Adaptive Immune Responses and induces long-term protection against disease”

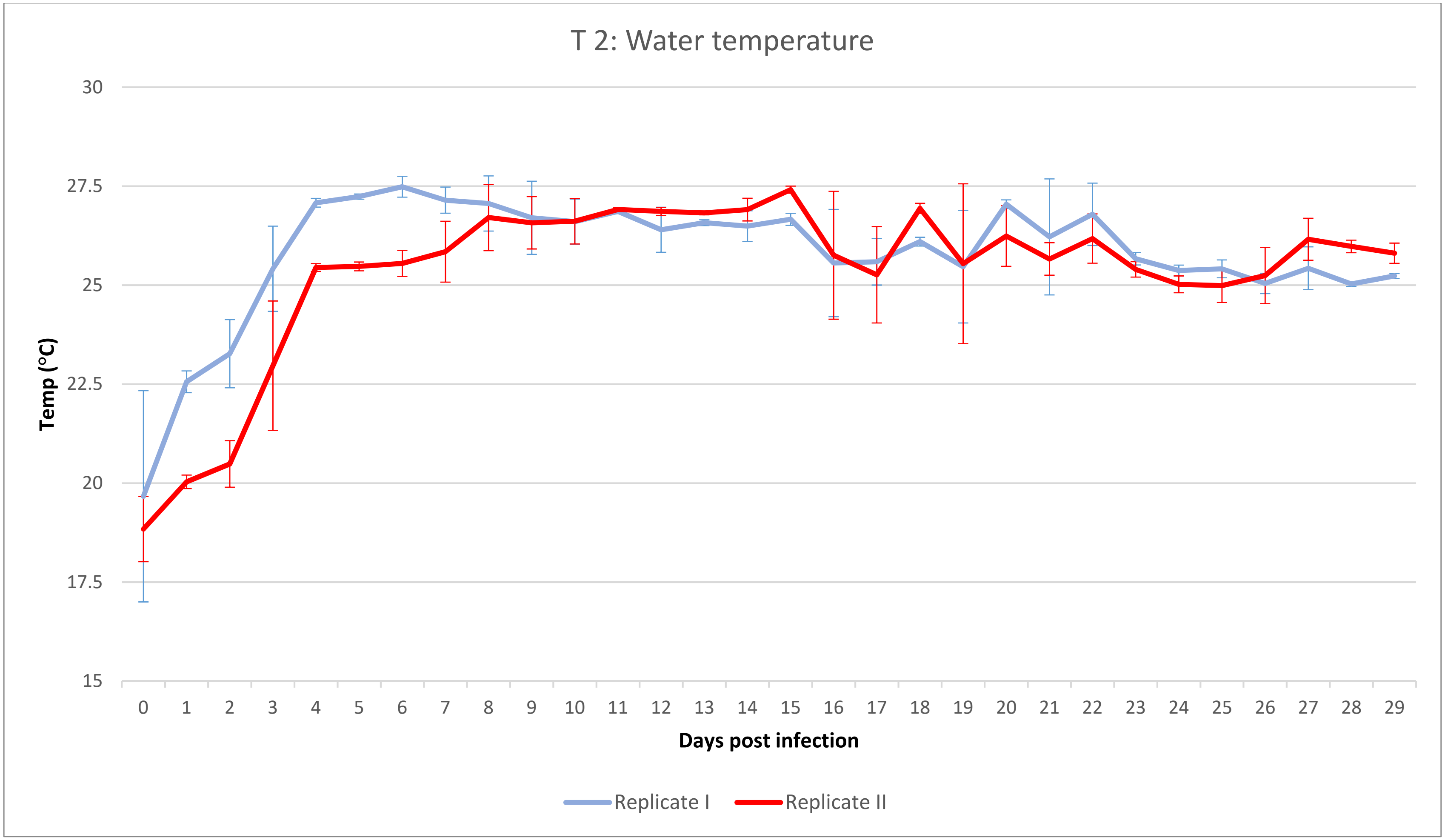


*Figure S4: Temperature curve for replicates in T2-challenge. The temperature was recorded every 10th minute during the entire experiment, in total 143-144 recordings pr. experiment day. The average temperature/day and the SD was calculated and plotted in the graph above. The replicate numbers refer to the replicates in table 2 in the paper and the colors are similar to the colors in the survival graph in figure 4.*

Supplementary figures to Barsøe et al 2021 “Nervous Necrosis Virus-like Particles (VLP) vaccine stimulates European Sea Bass Innate and Adaptive Immune Responses and induces long-term protection against disease”

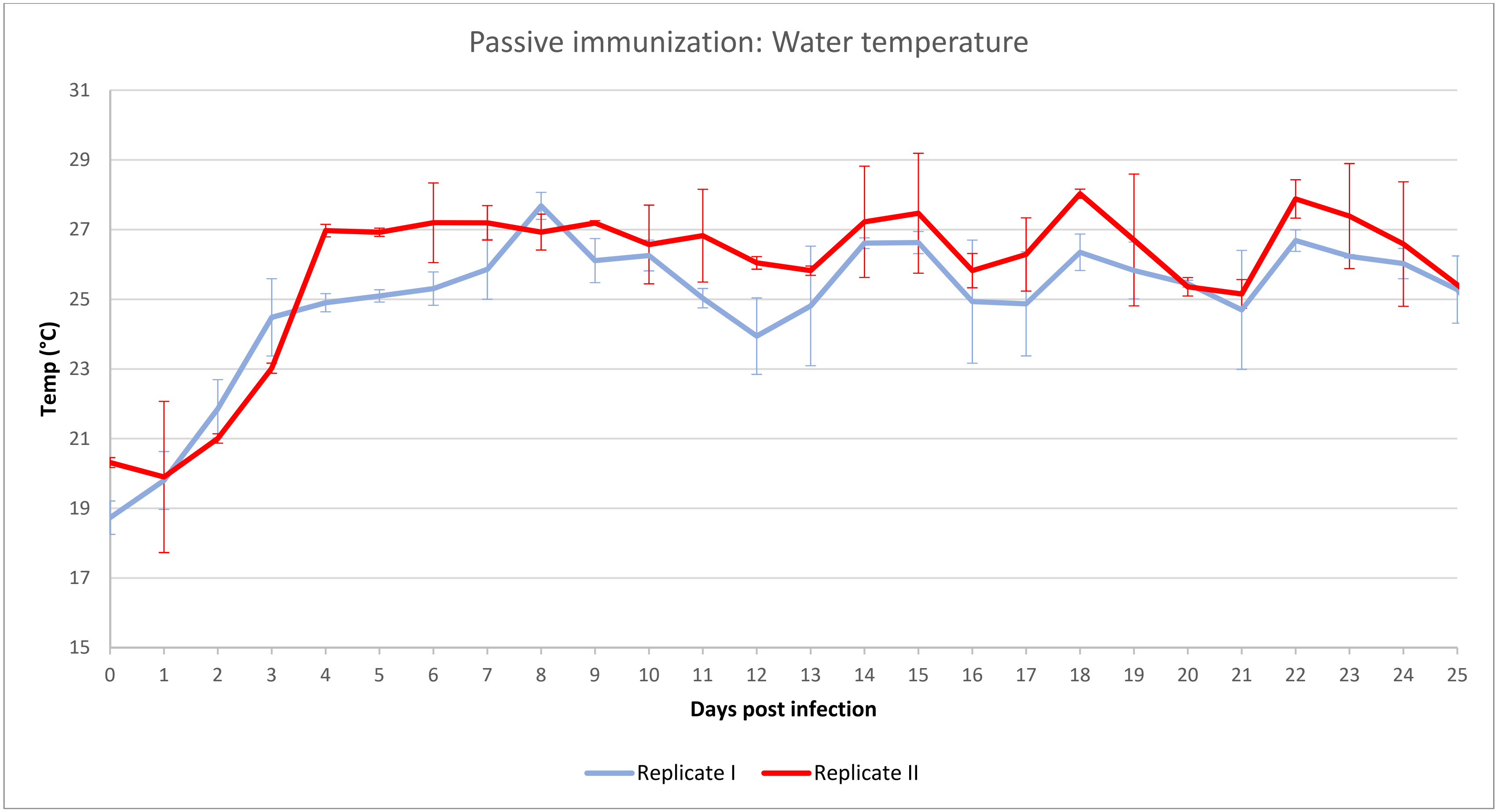


*Figure S5: Temperature curve for replicates in passive immunization. The temperature was recorded every 10th minute during the entire experiment, in total 143-144 recordings pr. experiment day. The average temperature/day and the SD was calculated and plotted in the graph above. The replicate numbers refer to the replicates in table 6 in the paper and the colors are similar to the colors in the survival graph in figure 11.*
