# Supplementary material for: Nervous Necrosis Virus-like Particle (VLP) Vaccine Stimulates European Sea Bass Innate and Adaptive Immune Responses and Induces Long-Term Protection against Disease
Source: Pathogens. 2021 Nov 12;10(11):1477. doi: 10.3390/pathogens10111477 (PMC8623669; doi:10.3390/pathogens10111477)
Supplement: Supplementary file 1 [file pathogens-10-01477-s001.zip › Table S1.pdf]

Supplementary material to Barsøe et al 2021 "Nervous Necrosis Virus-like Particles (VLP) vaccine stimulates European Sea Bass Innate and Adaptive Immune Responses and induces long-term protection against disease"

Table S1. Record of mortality and weight after vaccination and challenge.

Time	Status	PBS	VLP	COM
		**Mortality**		
**Day 1-21**	After vaccination	3/164 (1.8%)	0/165 (0%)	2/164 (1.2%)
		**Weight (g) [95% CI]**		
**T1**	Survivors	14.8 [12.0 – 17.5] (n = 26)	17.6 [16.1 – 19.1] (n = 51)	13.8 [11.3 – 16.3]* (n = 36)
**T2**	Diseased	40.5 [35.4 – 45.6] (n = 32)	34.6 [20.8 – 48.5] (n = 5)	36.3 [29.4 – 43.2] (n = 39)
	Survivors	48.1 [35.6 – 52.1] (n = 11)	56.2 [50.9 – 61.4] (n = 48)	38.8 [28.2 – 49.6]* (n = 15)

Mortality the first 21 days after vaccination and mean weight (g) [95% CI] after challenge. The fish were weighed individually either when terminating the experiment (survivors) or when euthanizing moribund fish (diseased). T1 = challenge 3 months pv., T2 = challenge 7.5 months pv., VLP = Virus-like particle vaccine, COM = commercial vaccine, PBS = phosphate buffered saline. * indicate significantly different from the mean of VLP vaccinated fish at the same time point/status (line) tested with ANOVA ($p<0.05$).
